# Protozoa-enhanced conjugation frequency alters the dissemination of soil antibiotic resistance

**DOI:** 10.1093/ismejo/wraf009

**Published:** 2025-01-27

**Authors:** Chenshuo Lin, Li-Juan Li, Kai Yang, Jia-Yang Xu, Xiao-Ting Fan, Qing-Lin Chen, Yong-Guan Zhu

**Affiliations:** State Key Laboratory for Ecological Security of Regions and Cities, Ningbo Urban Environment Observation and Research Station, Institute of Urban Environment, Chinese Academy of Sciences, 1799 Jimei Road, Xiamen 361021, China; Zhejiang Key Laboratory of Urban Environmental Processes and Pollution Control, CAS Haixi Industrial Technology Innovation Center in Beilun, Ningbo 315830, China; State Key Laboratory for Ecological Security of Regions and Cities, Ningbo Urban Environment Observation and Research Station, Institute of Urban Environment, Chinese Academy of Sciences, 1799 Jimei Road, Xiamen 361021, China; Zhejiang Key Laboratory of Urban Environmental Processes and Pollution Control, CAS Haixi Industrial Technology Innovation Center in Beilun, Ningbo 315830, China; State Key Laboratory for Ecological Security of Regions and Cities, Ningbo Urban Environment Observation and Research Station, Institute of Urban Environment, Chinese Academy of Sciences, 1799 Jimei Road, Xiamen 361021, China; State Key Laboratory for Ecological Security of Regions and Cities, Ningbo Urban Environment Observation and Research Station, Institute of Urban Environment, Chinese Academy of Sciences, 1799 Jimei Road, Xiamen 361021, China; University of Chinese Academy of Sciences, 19A Yuquan Road, Beijing 100049, China; State Key Laboratory for Ecological Security of Regions and Cities, Ningbo Urban Environment Observation and Research Station, Institute of Urban Environment, Chinese Academy of Sciences, 1799 Jimei Road, Xiamen 361021, China; State Key Laboratory for Ecological Security of Regions and Cities, Ningbo Urban Environment Observation and Research Station, Institute of Urban Environment, Chinese Academy of Sciences, 1799 Jimei Road, Xiamen 361021, China; Zhejiang Key Laboratory of Urban Environmental Processes and Pollution Control, CAS Haixi Industrial Technology Innovation Center in Beilun, Ningbo 315830, China; State Key Laboratory for Ecological Security of Regions and Cities, Ningbo Urban Environment Observation and Research Station, Institute of Urban Environment, Chinese Academy of Sciences, 1799 Jimei Road, Xiamen 361021, China; Zhejiang Key Laboratory of Urban Environmental Processes and Pollution Control, CAS Haixi Industrial Technology Innovation Center in Beilun, Ningbo 315830, China; State Key Laboratory of Urban and Regional Ecology, Research Center for Eco-Environmental Sciences, Chinese Academy of Sciences, Beijing 100085, China

**Keywords:** conjugation, antibiotic resistance genes (ARGs), protozoa, plasmids, virulence factors (VFS)

## Abstract

Protozoa, as primary predators of soil bacteria, represent an overlooked natural driver in the dissemination of antibiotic resistance genes (ARGs). However, the effects of protozoan predation on ARGs dissemination at the community level, along with the underlying mechanisms, remain unclear. Here we used fluorescence-activated cell sorting, qPCR, combined with metagenomics and reverse transcription quantitative PCR, to unveil how protozoa (*Colpoda steinii* and *Acanthamoeba castellanii*) influence the plasmid-mediated transfer of ARGs to soil microbial communities. Protozoan predation reduced the absolute abundance of plasmids but promoted the expression of conjugation-associated genes, leading to a 5-fold and 4.5-fold increase in conjugation frequency in the presence of *C. steinii* and *A. castellanii*, respectively. Excessive oxidative stress, increased membrane permeability, and the provoked SOS response closely associated with the increased conjugative transfer. Protozoan predation also altered the plasmid host range and selected for specific transconjugant taxa along with ARGs and virulence factors carried by transconjugant communities. This study underscores the role of protozoa in the plasmid-mediated conjugative transfer of ARGs, providing new insights into microbial mechanisms that drive the dissemination of environmental antibiotic resistance.

## Introduction

Antibiotic resistance poses substantial threats to human health, with an estimated 1.27 million deaths directly attributable to antibiotic-resistant bacterial infections in 2019, and up to 10 million deaths expected by 2050 [1]. Worryingly, antibiotic resistance genes (ARGs) often co-occur and co-transfer with virulence factor genes (VFGs) that help bacteria to invade humans/animals and increase their disease-causing capacity [2]. This coexisting will increase the risk of pathogenic antibiotic-resistant bacteria because they can both cause disease and invalidate antibiotic therapy [3]. The rapid spread of environmental ARGs, VFGs, and the emergence of pathogenic antibiotic-resistant bacteria are largely attributed to horizontal gene transfer (HGT) [4]. Plasmid conjugation, a principal mechanism of HGT, facilitates the transfer of ARGs from donor to recipient microorganisms through direct cell-to-cell contact [3]. Plasmids function as vehicles can transfer accessory genes such as ARGs and VFGs between bacteria and even pathogens, including those distantly related species across phyla and even domains [5]. Consequently, plasmid-mediated conjugation plays a critical role in the maintenance and spread of ARGs and VFGs by promoting gene shuffling of genes within microbial communities [6].

The emergence, evolution, and dissemination of antibiotic resistance and virulence factors are natural phenomena, essential for the survival and adaptation of bacteria, driven by factors such as predation, energy substrate warfare, and other environmental pressures [7–9]. Soil acts as both a significant source and reservoir of ARGs and a diverse spectrum of microorganisms [10, 11]. Protozoa, as a non-negligible component of soil microorganisms, are the main predators of bacteria. The predation pressure exerted by protozoa is recognized as a robust biotic force within bacterial communities, driving the evolution and spread of ARGs [12, 13]. Protozoa encapsulate bacteria within ciliate vacuoles, where both ARG donors and recipients are concentrated, thereby enhancing the frequency of HGT events through intensive cellular contact. Protozoa (*Tetrahymena thermophilaha*) have been reported to increase the persistence and spread of the antibiotic resistance plasmid RP4 in populations of the opportunistic pathogen *Serratia marcescens* [14]. Additionally, Matsushita reported that *Tetrahymena* promoted the conjugation frequency of a bla_IMP-1_-encoding IncA/C plasmid between *Escherichia coli* and *Aeromonas caviae* [15]. However, HGT promoted by protozoa is primarily observed in endosymbiotic relationships with select bacterial model strains in pure culture experiments, potentially inadequately representing the true impact of protozoan predation on the dissemination of ARGs in the environmental microbial communities. Furthermore, protozoa exert top-down regulation on microbiome composition, population dynamics, and functional traits, thereby conferring a selective advantage to bacterial communities possessing specific grazing-resistant traits [16, 17]. The response of highly diverse soil microbial communities to protozoan predation may vary widely. However, the influence of protozoa on the composition and characteristics of transconjugants, as well as the susceptibility of specific bacterial communities to protist predation pressure, remains inadequately understood. This ambiguity underscores several critical inquiries: (i) to what extent do protozoa influence the fate and spread of plasmids in microbial communities, (ii) which bacteria are likely to acquire plasmid-borne ARGs under protozoan predation, and (iii) what are the mechanisms by which protozoa influence HGT at the community level.

**Figure 1:**
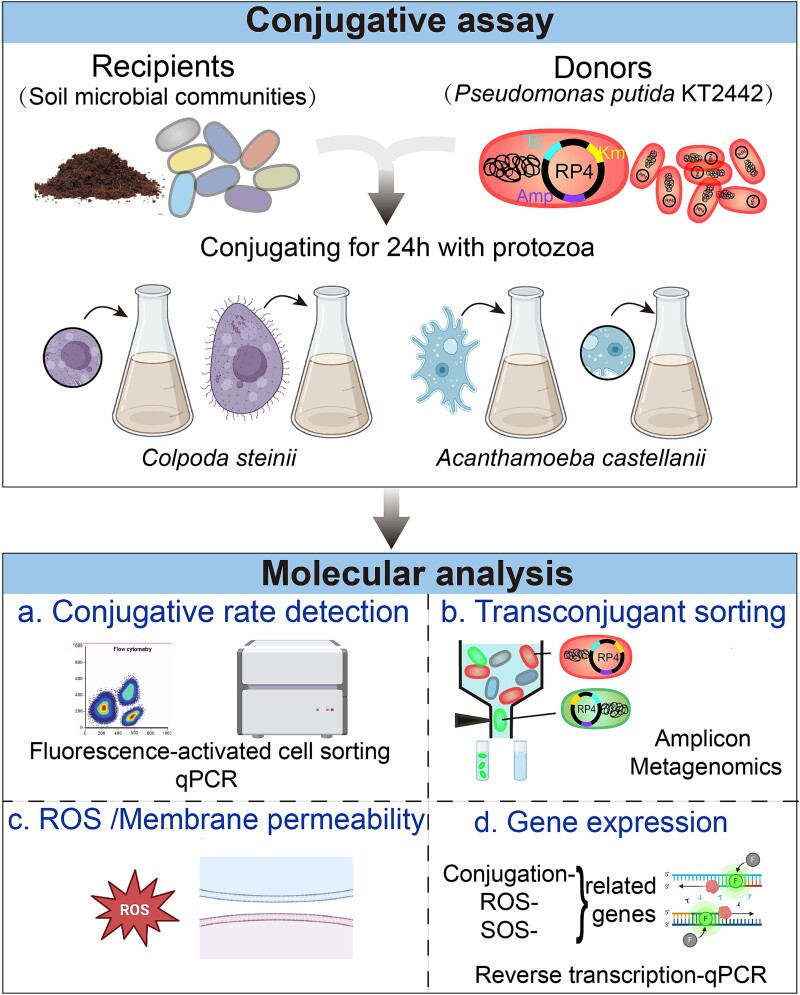
Experimental design of this study. Firstly, fluorescently dsred-tagged *Pseudomonas putida* hosting the plasmid RP4 was added to the extracted soil community in mating assays exposing to different protozoan trophozoites or cysts (*Colpoda steinii* and *Acanthamoeba castellanii*). The RP4 plasmid hosts multiple resistance genes against ampicillin, kanamycin, and tetracycline, and was here used in a *gfp*-tagged form. The mating assays were incubated at 28°C for 24 h. secondly, the conjugative transfer frequency was calculated by a fluorescence microscopy, followed by fluorescence-activated cell sorting of transconjugants. Subsequently, 16S rRNA gene amplicon and metagenomic sequencing were employed to characterize the composition, ARGs and VFs profiles of transconjugant communities upon exposure to different protozoa. Then we detected the level of reactive oxygen species and membrane permeability. Finally, the expression of genes associated with conjugation, reactive oxygen species, and SOS response was detected using reverse-transcriptase quantitative PCR.

We selected two typical soil protozoa, *Colpoda steinii* and *Acanthamoeba castellanii,* to assess the influence of protozoa on the conjugative transfer of ARGs carried by plasmids in soil microbial communities. A broad-host-range green fluorescence-tagged IncP-*α* RP4 conjugative plasmid carrying three ARG subtypes was introduced to the extracted soil microbial community via a red fluorescence-tagged *Pseudomonas putida* KT2442 strain. Fluorescence-activated cell sorting (FACS) and qPCR were applied to quantify the plasmid fate and the conjugation frequency of plasmid-borne ARGs under different protozoan predation pressures. Additionally, high-throughput 16S rRNA gene amplicon sequencing and metagenomics characterized the community composition and molecular traits (ARGs and VFGs) of potential host range receiving plasmid-borne ARGs in response to different predation stress. The underlying influencing mechanism of protozoa on plasmid-mediated conjugation was further revealed by measuring the transcriptional response of the soil microbial community by reverse-transcriptase quantitative PCR (RT-qPCR) (Fig. 1).

## Materials and methods

### Protozoa

Two protozoa from agricultural soil with distinct feeding and movement modes were used: filter-feeding protozoa *C. steinii* exhibiting a higher predation rate and surface-feeding protozoa *A. castellanii*. Both species are widely distributed and abundant soil protozoa serving as key microbial predators in the soil micro-food web. Detailed information for protozoan growth is provided in the Supplementary Material.

### Donor strain and recipients

The donor strain, *P. putida* KT2442, carries the broad host range conjugative IncP plasmid RP4, which contains ARGs against kanamycin (Km), ampicillin (Amp), and tetracycline (Tc) [18]. The RP4 plasmid is tagged with a green fluorescent protein (*gfp*) gene under a *lacI^q^* repressible promoter [19]. The donor strain exhibits red fluorescence due to chromosomal tagging with *dsRed* and a constitutively expressed *lacI^q^*. The donor strain was grown in LB broth with antibiotics (100 μg/ml Amp, 50 μg/ml Km, and 20 μg/ml Tc). Soil microbial communities (recipient) were extracted from a vegetable field in Xiamen, China (24°64′N, 118°05′E). More information about recipients is provided in the Supplementary Material.

### Conjugation experiments

Conjugation assays were conducted by mixing the soil microbiome with donor *P. putida* KT2442 (1:1, v/v) under different protozoan predation pressure in sterile PAS buffer. Donors and recipients were adjusted to 5 × 10^7^ CFU/ml with PBS. Approximately 5 × 10^3^ cells ml^−1^  *C. steinii* and 10^4^ cells ml^−1^  *A. castellanii,* either trophozoites or cysts, were added to each microcosm and then incubated at 28°C for 24 h, respectively. Control included groups without protozoa or with heat-killed protozoa (90°C water bath for 10 min). To obtain protozoan cysts, both *C. steinii* and *A. castellanii* were starved in sterile PAS for one week and observed with an inverted microscope. To validate the contribution of reactive oxygen species (ROS) in enhanced conjugation under protozoa predation, a set of conjugation assays were performed in the addition of a ROS scavenger—thiourea (100 μM). To determine the transfer frequency of both free-living and intracellular bacteria, Triton X-100 was added to a final concentration of 1% to lyse protozoa and release internalized bacterial cells [20].

### Transconjugants sorting, amplicon sequencing, and qPCR

Transconjugants were sorted using a S3e Cell Sorter (Bio-Rad). The donor fluorescence signal was excited by 561 nm and checked in 586/25 nm for *dsRed*. The GFP fluorescence detection was based on excitation at 488 nm laser with emission at 525/30 nm. *Escherichia coli* K12 and *E. coli* K12 carrying RP4::*gfp* functioned as fluorescence-negative and gfp-positive control, respectively. Compared with control cells and donors, transconjugants were sorted according to higher green fluorescence intensity and lower red fluorescence intensity (Fig. S1). After 24 h, a minimum of 100 000 targeted transconjugant cells were collected under the purity mode with four biological replicates. The gel electrophoresis results confirmed the accuracy of transconjugants that all sorted transconjugants harbored *gfp*, *blaTEM*, and *tetA* genes but no *dsRed* gene (Fig. S2A-D). Primer and amplification conditions are provided in the Supplementary materials (Table S1). The DNA of the sorted transconjugants was extracted using the FastDNA Spin Kit for Soil (MP Biomedical) [21]. The V4 region of the 16S rRNA genes was amplified using primer set 515F/806R [22]. The 16S rRNA gene amplicon sequencing was performed on the MiSeq PE250 sequencing platform (Illumina). Sequences were processed using the QIIME2 pipeline (https://qiime2.org) [23]. SILVA (v138.1) [24] database was used for taxonomic identification of bacteria.

To verify the predation pressure of protozoa to the bacterial communities, we quantified the absolute abundance of total bacteria, donor strain, and RP4 plasmid by qPCR using the same 16S rRNA, *dsRed*, and *gfp* gene primers as for PCR, respectively. The detail information of the qPCR assays was available in Supplementary method. The copy number of transconjugants was calculated by Copies*_gfp_* - Copies*_dsRed_*. Therefore, the transfer frequency of RP4 plasmids was calculated by following formula using qPCR results:


(1) \begin{equation*} \mathrm{Conjugants}\ \mathrm{per}\ \mathrm{recipient}=\frac{\ \mathrm{C}{opies}_{\mathrm{g} fp}-\mathrm{C}{opies}_{\mathrm{d} sRed}}{\frac{\mathrm{C}{opies}_{16\mathrm{s}\ rRNA}}{4.1}-\mathrm{C}{opies}_{\mathrm{d} sRed}} \end{equation*}

In Eq. 1, the average number of 16S rRNA genes per bacterial cell is estimated to be 4.1 according to the Ribosomal RNA Database [25]. Thus, copies_16S rRNA_ /4.1- copies *_dsRed_* presents the copies of potential recipients. The ratio of donor to recipient was calculated by following formula using qPCR results:


(2) \begin{equation*} \mathrm{Donors}\ \mathrm{per}\ \mathrm{recipient}\ \left(\mathrm{D}/\mathrm{R}\right)=\frac{\ \mathrm{C}{opies}_{\mathrm{d} sRed}}{\frac{\mathrm{C}{opies}_{16\mathrm{s}\ rRNA}}{4.1}-\mathrm{C}{opies}_{\mathrm{d} sRed}} \end{equation*}

### Multiple displacement amplification and metagenomic sequencing analysis

The genomic DNA of transconjugants was amplified by multiple displacement amplification (MDA) using REPLI-g Midi kit (QIAGEN). MDA reaction was incubated at 30°C for 8 h, then inactivated at 65°C for 3 min. MDA products served as templates for screening using bacterial 16S rRNA gene primers of 515F and 806R. PCR products were detected by an agarose gel electrophoresis. Negative controls with sterile water were processed in parallel and resulted in no PCR products. A HiSeq System (Illumina) was used to perform the metagenomic sequencing at the Majorbio Bio-Pharm Technology Co., Ltd., yielding 15 Gb of sequence data for each sample.

Sequencing reads were quality trimmed using fastp to remove sequences less than 50 bp, containing ambiguous bases, or average quality scores lower than 30 [26]. Sequences detected in negative control samples were subsequently filtered using fastp. Gene sequences were analyzed using DIAMOND against the Kyoto Encyclopedia of Genes and Genomes (KEGG) database to identify ecologically relevant functional subsystems [27]. ARG-like sequences in clean reads were annotated by ARGs-OAP (v2.0) [28] with default settings. The filtered sequences were assembled using MEGAHIT (v1.1.3) [29] with default parameters and were assessed using QUAST (v 5.0.2) [30]. The open reading frames for each assembled contig were predicted using Prodigal v.2.6.3 [31]. Predicted gene sequences were then clustered using MMseqs2 [32] to obtain unique gene sets. Virulence factors (VFs) were aligned to the virulence factors of pathogenic bacteria database database [33] (only experimentally validated), using BLASTn with the E-value ≤10^−10^, similarity, and query coverage≥80%. ICEfinder was used to identify mobile genetic elements, including integrative and conjugative elements and integrated and mobilizable elements [34].

### ROS and cell membrane permeability

ROS production and cell membrane permeability of donor and recipient communities were assessed using a Guava EasyCyte Flow cytometer, according to previous studies [35, 36]. The donor *P. putida* KT2442 and the recipient soil microbial community were each diluted to a concentration of 10^6^ cells/ml. To measure ROS production, the donor and recipient samples were stained with 2′,7′-dichlorofluorescin diacetate dye (20 μM) and incubated separately in the dark for 30 minutes. Following staining, donor and recipient were incubated with either *C. steinii* (10^3^ cells) or *A. castellanii* (5 × 10^3^ cells) at 28°C for 4 h in the dark, respectively. Solvent DMSO and H_2_O_2_ served as the negative and positive controls, respectively. Cell membrane permeabilities were evaluated using fluorescent staining with PI and Syto9. Boiled conjugation mixtures stained with PI, conjugation mixtures stained with SYTO9, conjugation mixtures without PI and SYTO9 were PI-positive controls, SYTO9-positive controls, and negative controls, respectively.

### Reverse transcriptase-quantitative PCR

Previous studies have shown that protozoa promote the expression of integron-integrase in *Vibrio cholerae* by triggering ROS and SOS response, which is associated with conjugation transfer [13]. We therefore hypothesized that protozoa-induced ROS and SOS response might also influence conjugative frequency. To test this hypothesis, the expression of conjugation- (*trbB*, *trfA*, *traJ*), ROS- (*ahpC*, *trxB*, *aphF*) and SOS-related genes (*recA*, *lexA*, *mutS*) were quantified with RT-qPCR. After co-culturing donor strains (5 × 10^7^ CFU), recipient bacteria (5 × 10^7^ CFU) with *C. steinii* (5 × 10^3^ cell), *A. castellanii* (1 × 10^4^ cell), and protozoa-free PAS solution for 4 h, respectively, total RNA was extracted using a QIAGEN RNeasy mini kit. RNA was then reverse transcribed into cDNA using a HiScript Ill All-in-one RT SuperMix Perfect for qPCR (Vazyme). RT-qPCR was conducted using a SYBR Green I approach (TaKaRa) on a Roche 480 system (LightCycler480II) using the comparative Ct (ΔΔCt) method. The 16S rRNA gene was the reference gene [37]. Primers and reaction conditions for RT-PCR assays are listed in Table S2. Considering that the proportion of plasmid-carrying cells in the mixture might influence expression of conjugation associated genes, we normalized the expression of conjugation-associated genes with the number of plasmid-containing strains using the below equations:


(3) \begin{equation*} \mathrm{gene}\ \mathrm{expression}\ \mathrm{per}\ \mathrm{plasmid}=\frac{\ gene\ expression}{\frac{copies_{gfp}}{copies_{16S\ rRNA}/4.1}} \end{equation*}

### Statistical analysis

The transconjugant communities from different treatments were visualized by non-metric multidimensional scaling (NMDS). Nonparametric methods Wilcoxon rank-sum test was used to assess compositional homogeneity among the groups using SPSS 20.0 (IBM). The similarities of transconjugant communities were analyzed with ANOSIM in the R 4.3.2 with the vegan package [38]. Linear discriminant analysis (LDA) of effect size (LEfSe) was performed to identify indicator bacterial taxa in transconjugants communities that significantly change in different treatments with adjusted *P* < 0.05 and LDA score > 3 [39]. A bacterial human pathogen database containing 684 16S rRNA full-length sequences and a multiple bacterial pathogen detection (MBPD) database [40] were used to identify the potential pathogen in transconjugant communities at the e-value <1 × 10^−5^ and > 99% identity threshold level, as previously described [41]. STAMP analyses and Venn diagrams were analyzed using OmicStudio tools (https://www.omicstudio.cn/tool/) [42]. Functional profiles and potential metabolic functions of transconjugants were predicted using functional annotation of prokaryotic taxa (FAPROTAX) based on 16S rRNA gene amplicon sequencing [43] and PICRUSt2 [44], respectively.

## Results

### The fate of PR4 plasmids and conjugation frequency

**Figure 2:**
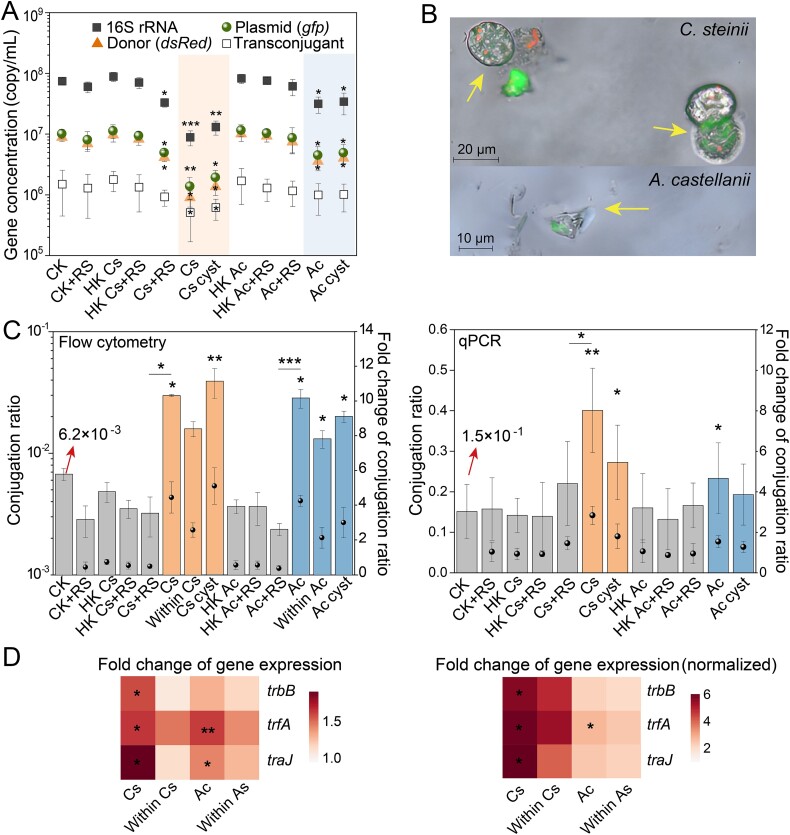
The fate and conjugative transfer of the RP4 plasmids from *P. putida* KT2442 to soil microbial communities with protozoa. (A) The concentration of 16S rRNA, *dsRed*, *gfp* genes and potential transconjugants in conjugation pools after 24 h co-incubation*.* (B) Epifluorescence images displaying RP4 host cells and donors engulfed by *C. steinii* and *A. castellanii* and depicting. (C) Different methods showing the conjugative transfer frequency (left axis) with protozoa and its fold changes (right axis) compared to the protozoa-free group after 24 h (n = 4). Conjugative transfer frequency was estimated in each treatment including protozoa-free (CK), heat-killed protozoa (HK Cs and HK Ac), protozoa trophozoites (Cs and Ac), protozoa cyst (Cs cyst and Ac cyst) and bacteria within protozoa (within Cs and within As), and + RS indicates the addition of 100 mM of the ROS scavenger, thiourea. (D) Fold changes of expression of *trbB*, *trfA*, and *traJ* genes in mixture systems with protozoa compared to the protozoa-free group and the fold change after normalization (normalizing it to the number of plasmids bearing strains present in the mixture) (n = 6). Statistical significance between each protozoan treatment and protozoa-free group was determined using ANOVA and depicted with ^*^P < 0.05 and ^*^^*^P < 0.01.

The fate of bacterial communities, donor strains, and RP4 plasmids decreased after 24 h of co-incubation with the two protozoa whether either trophozoites or cysts (*P* < 0.05, Fig. 2A). The average copy number of the 16S rRNA, *gfp*, and *dsRed* genes with *C. steinii* were about an order of magnitude lower than those in the protozoa-free group. There was no significant difference in the impact of cysts and trophozoites on the copy number of these three genes. The D/R showed no significant differences across treatments (Fig. S3). The presence of protozoa also decreased the potential abundance of transconjugants in the community compared to the group without protozoa, especially *C. steinii* (*P* < 0.05, Fig. 2A). Fluorescent microscopy further revealed the presence of both donors and transconjugants carrying GFP-expressing RP4 plasmids within protozoa (Fig. 2B).

The presence of *C. steinii* and *A. castellanii*, whether as cysts or trophozoites, led to marked increases in the conjugation frequencies. Conjugation frequencies with *C. steinii* and *A. castellanii* trophozoites measured using flow cytometry were approximately 2.98 × 10^−2^ and 2.85 × 10^−2^ per recipient, respectively, representing 5 and 4.5 times the frequency observed in the protozoa-free group (~6.72 × 10^−3^, *P* < 0.05; Fig. 2C). Similarly, the presence of protozoan cysts significantly increased the conjugation frequency in the mixture compared to the protozoa-free group (*P* < 0.05). The qPCR also showed increased conjugation frequencies with two protozoa compared to the protozoa-free group (*P* < 0.05, Fig. 2C). The average transconjugants per recipient with *C. steinii* and *A. castellani* were 0.38 and 0.23, respectively, representing increases of 2.7-fold and 1.5-fold compared to the protozoa-free group (Fig. 2C).

At the RNA transcription level, RT-qPCR results showed that in the presence of *C. steinii* trophozoites, transcription of conjugation-associated genes in the mixture system (*trbB*, *trfA*, and *traJ*) increased by an average of 2.1-, 1.8-, and 4.3-fold, respectively, after 4 h (Fig. 2D; *P* < 0.05). Treatment with *A. castellanii* trophozoites resulted in an average 1.3-, 1.5-, and 1.4-fold increase in *trbB*, *trfA*, and *traJ* gene transcription compared to the protozoa-free group, respectively (Fig. 2D). Similarly, the expression of all conjugation-associated genes per plasmid-carrying cell was higher in the protozoa-treated groups than in the protozoa-free group, especially for *C. steinii* trophozoites (Fig. 2D; *P* < 0.05).

### Protozoa selected for specific transconjugant communities

**Figure 3:**
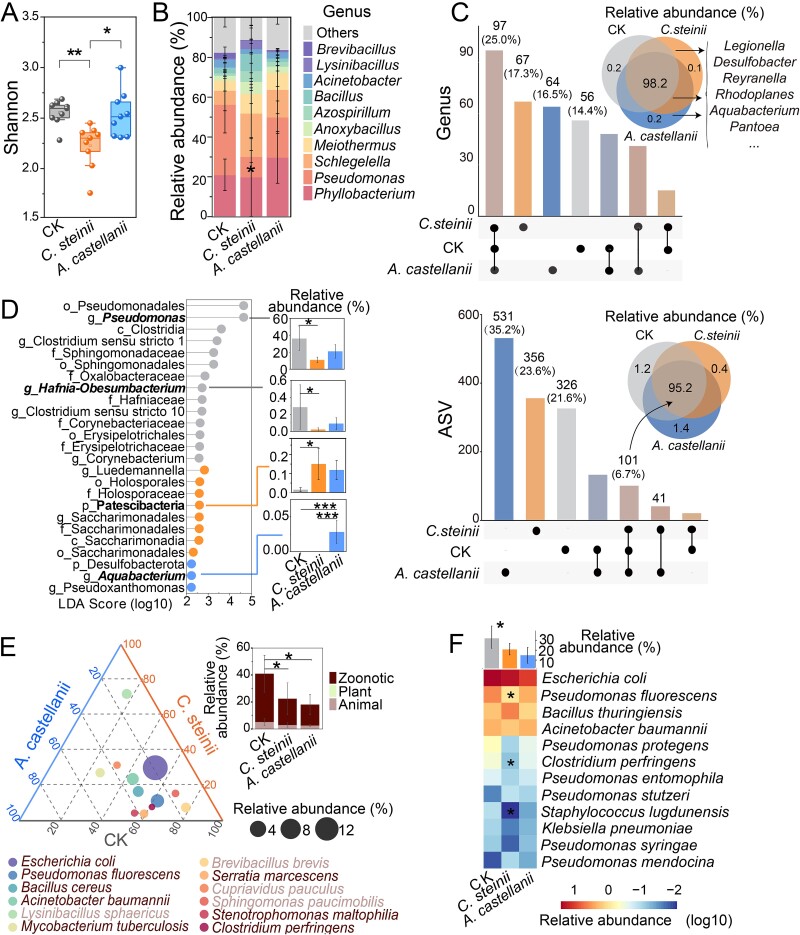
Community and molecular characteristics of transconjugant pools. (A) Alpha-diversity (Shannon index) and (B)community compositions at genus level of the transconjugant communities (n = 4). The relative abundance of *Pseudomonas* transconjugants in *C. steinii* group was significantly lower than that of the control group (^*^P < 0.05) (C) Venn diagrams showing the number and the relative abundance of shared and unique genus or ASVs in different transconjugant pools using subsampling data. (D) LEfSe analysis identifies biomarkers of transconjugant communities with their relative abundance under different protozoan predations (α > 0.05 LDA score > 2.0). (E) The relative abundance of the top 12 potential pathogens found in transconjugant communities with different protozoa based on MBPD and (F) self-constructed human pathogen database. The column plot above the heatmap represents the average total relative abundance of potential pathogens in corresponding groups. Asterisks indicate the potential pathogens in transconjugant communities of whose relative abundance showed significant changes under protozoan exposure compared to the control group (^*^P < 0.05).

After 24 h of mating, the *C. steinii* treatment group exhibited the lowest alpha diversity of transconjugants (Fig. 3A; *P* < 0.05), whereas no statistically significant difference was observed between the protozoa-free and the *A. castellanii* group. NMDS analysis showed that the distribution of transconjugant communities was not significantly different in the presence or absence of protozoa (Fig. S4A). Dominant phyla and genera of the transconjugant community remained consistent across treatments. *Proteobacteria* was the most abundant phylum, accounting for 76.3% - 83.8%, followed by *Firmicutes* (7.9% - 14.0%) and *Deinococcota* (Fig. S5). At the genus level, *Phyllobacterium*, *Pseudomonas*, and *Schlegelella* were the most abundant across all transconjugant pools (Fig. 3B). The proportion of *Pseudomonas* was significantly lower in *C. steinii* transconjugants than other groups (Fig. 3B, *P* < 0.05). Most phyla (Fig. S6) and genera were shared across protozoa and protozoa-free treatments, with the relative abundance of shared genera accounting for 98.2% of the total sequences (Fig. 3C). Several genera, including *Legionella*, *Desulfobacter*, *Rhodoplanes, Reyranella*, and *Aquabacterium* (in *A. castellanii* group only) were observed exclusively in transconjugants with protozoa (Fig. 3C). These genera except *Aquabacterium* were found in more than half of the samples from each protozoan treatment, with average abundances of < 0.02% for each genus*.* At the ASV level, the number of unique ASVs for each group was three times higher than shared ASVs, especially for *A. castellanii* group. However, the relative abundance of unique ASVs was all less than 1.5%. LEfSe analysis further revealed unique biomarkers for each sorted transconjugant community, such as *Pseudomonas, Hafnia-Obesumbacterium,* and *Clostridium sensu stricto 10* for the protozoa-free group, *Patescibacteria* for the *C. steinii* group, and *Aquabacterium* for the *A. castellanii* group, all significantly enriched in their respective transconjugants (Fig. 3D, *P* < 0.05). Additionally, the average relative abundance of all significantly enriched protozoa associated biomarkers accounted for < 1% of the total sequences. Despite no statistical differences in the potential functional structure of the transconjugant community (Fig. S4B), the relative abundance of some level 3 KEGG Orthologies, such as novobiocin biosynthesis and glucagon signaling pathway, varied across treatments (Fig. S7, *P* < 0.05).

We identified 52 (self-constructed databases) and 249 (MBPD) kinds of potential pathogens in all transconjugant communities (Table S3). The top 4 dominant potential pathogens in all transconjugant pools were: *E. coli*, *Pseudomonas fluorescens*, *Bacillus thuringiensis,* and *Acinetobacter baumannii* (Fig. 3E and F). Most potential pathogenic transconjugants were present across different treatments (Fig. S8A and S8B). The diversity of potential pathogenic transconjugants did not differ significantly between treatments with and without protozoa (Fig. S8C and S8D). The proportion of total potential pathogenic transconjugants, whether animal or zoonotic, was significantly lower in the presence of protozoa than in the protozoa-free group (Fig. 3E and F, *P* < 0.05; *P* < 0.05). Most dominant potential pathogens were present in the protozoa-free group (Fig. 3E). The relative abundance of *P. fluorescens* and *Clostridium perfringens* in transconjugants exposed to *C. steinii* was significantly lower than those in the protozoa-free group (Fig. 3F, *P* < 0.05; Fig. S9, *P* < 0.05). Additionally, FAPROTAX analysis indicated that the relative abundance of potential bacterial human pathogens and bacteria associated with human guts was lower in transconjugants with *C. steinii* than the protozoa-free group (Fig. S10, *P* < 0.05).

### Protozoa selected for ARGs and VFs in transconjugants

**Figure 4:**
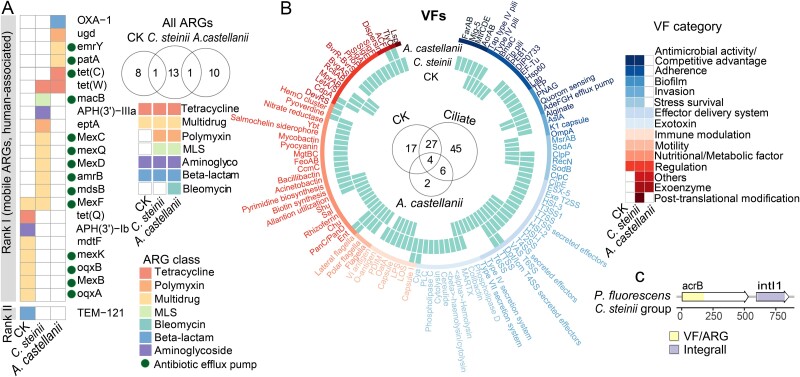
ARGs and VFs carried by transconjugant communities. (A) ARGs and (B) VFs found in transconjugant communities under different protozoan predation. (C) Schematic of the genetic organization of VFs co-occurring with MGEs from metagenome assemblies in transconjugant communities.

Protozoa not only influenced the community structure of transconjugant communities but also impacted the ARGs and VFs carried by transconjugants. Under different protozoan predation pressures, each transconjugant community carried distinct ARGs and VFs, with no shared ARGs and few shared VFs (Fig. 4A and B). A total of seven ARG classes including 34 subtypes were found in all transconjugant pools. Rank I high-risk ARGs and the multidrug-resistant ARGs class were detected in all transconjugant communities (Fig. 4A). *OpmH*, *MexF*, and *APH(3′)-Ib* subtypes were dominant in transconjugants from control, *C. steinii*, and *A. castellanii* treatments, respectively. ARGs belonging to Polymyxins, macrolide-lincosamide-streptogramin, and Bleomycin class were specifically carried by transconjugants under predation pressure (Fig. 4A). In terms of VFs, we detected a total of 13 categories with 101 VFs. *TlyC* (Exoenzyme), *Cya* (Exotoxin), Capsule I (Immunomodulatory), pyoverdine (Nutritional/Metabolic factor), ACF, and CdpA (Others) were only detected in transconjugants under both protozoan predation pressure (Fig. 4B). Moreover, the virulence factor gene *acrB,* which also function as the efflux pump, was found adjacent to the class 1 integron-integrase gene Intl1 in the transconjugant contig annotated *P. fluorescens* in the presence of *C. steinii* (Fig. 4C).

### Protozoa stimulated ROS generation, cell membrane permeability, and SOS response

**Figure 5:**
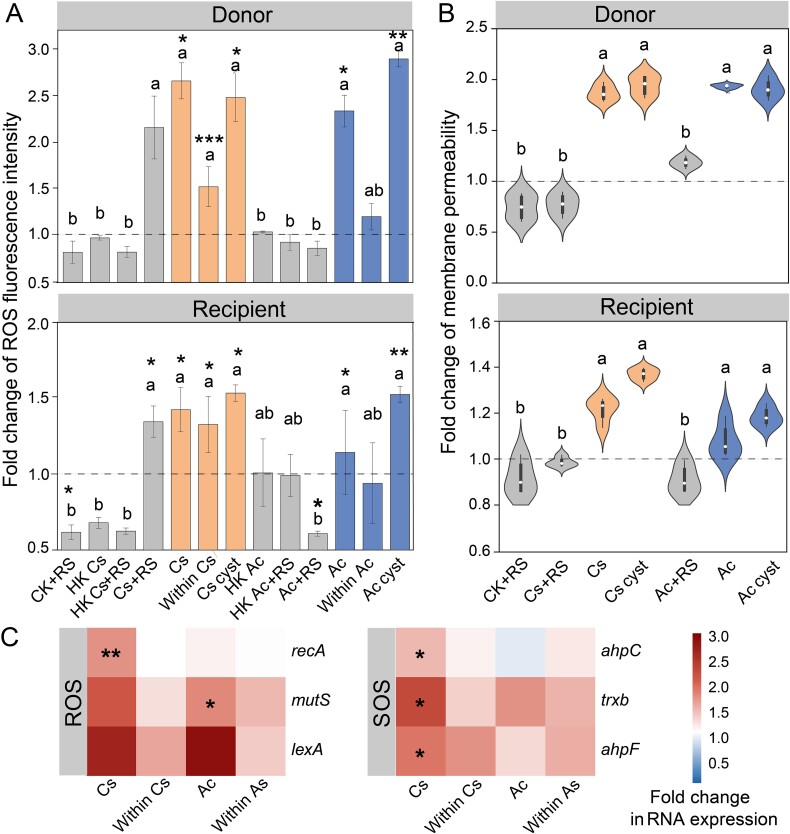
ROS generation, membrane permeability, and RNA transcriptional changes of ROS- and SOS-associated genes expression of transconjugants. (A) Fold changes of ROS fluorescence intensity of donor and recipient (n = 6). (B) Cell membrane permeabilities of donor and recipient communities in the presence and absence of protozoa (n = 6). (C) The expression of ROS- and SOS-associated genes (n = 6). Fold changes are all based on the control group (CK). Significant differences of ROS generation, membrane permeability, and RNA transcriptional between protozoa exposure groups and the control group were tested with ANOVA and shown with ^*^P < 0.05 and ^*^^*^P < 0.01. Different letters in the histograms and violin plots indicate significant differences between treatments by the nonparametric Kruskal–Wallis test.

Our study observed a significant induction of ROS production and changes in cell membrane permeability in microbial communities upon protozoan predation. Specifically, the presence of *C. steinii* and *A. castellanii*, either as cysts or trophozoites, led to marked increases in ROS levels after 4 h of exposure. ROS production in both donor and recipient communities was ~1.4- and 2.6-fold higher with *C. steinii* trophozoites than in the protozoa-free group, respectively (Fig. 5A; *P* < 0.05). Similarly, *A. castellanii* trophozoites elevated ROS levels by ~1.3- and 2.3-fold in both donor and recipient communities (Fig. 5A; *P* < 0.05). The addition of the ROS scavenger thiourea significantly reduced the conjugation frequency in the presence of *C. steinii* and *A. castellanii* trophozoites to levels comparable with heat-killed or protozoa-free groups, highlighting the role of ROS in facilitating plasmid transfer (Fig. 2C). Moreover, the introduction of protozoa led to a considerable increase in cell membrane permeability. The donor strain *P. putida* KT2442 showed 1.8- to 2.0-fold higher cell membrane permeability in the *C. steinii* group and 1.8- to 1.9-fold in the *A. castellanii* group compared to controls (Fig. 5B; *P* < 0.05). The recipient communities also demonstrated similar increases in membrane permeability due to protozoan predation (Fig. 5B).

To further elucidate the potential mechanism involved, RT-qPCR analysis was conducted to measure the transcription levels of ROS- and SOS-related genes. The expression of alkyl hydroperoxide reductase genes (*ahpC* and *ahpF*) and thioredoxin reductase (*trxB*) was significantly upregulated by 1.6-, 2.0-, and 1.9-fold, respectively, in the presence of *C. steinii* trophozoites (Fig. 5C; *P* < 0.05). Moreover, the transcription of the SOS response-related gene *recA* was increased by 3.9-fold under the predation pressure of *C. steinii* trophozoites (Fig. 5C; *P* < 0.01). The addition of *A. castellanii* showed a similar trend in gene expression elevation, these changes were not statistically significant.

## Discussion

Investigating the mechanisms by which protozoa influence the dissemination of ARGs at the community level is essential for understanding the natural drivers behind ARG dissemination [45]. Here, we studied how exposure to protozoa *C. steinii* and *A. castellanii* affects the conjugation frequency to RP4 plasmids and shapes the transconjugant community at the community level using FACS, qPCR, metagenomic sequencing, and RT-qPCR. We show that protozoan predation decreased the absolute abundance of RP4 plasmids but increased their conjugation frequency from *P. putida* KT2442 to soil bacterial communities. We observe the increased expression of conjugation-associated genes, accompanied by consistently elevated ROS levels, increased cell membrane permeability, and induction of the SOS response. Protozoa selectively enriched specific transconjugants, decreased the proportion of potential pathogenic transconjugants and affected the ARGs and VFs carried by transconjugant communities. These results underscore the pivotal role and mechanisms of protozoa in facilitating the plasmid-mediated dissemination of ARGs.

Protozoan predation pressure is typically reflected in a reduction of total bacterial biomass. In this study, both donor strains and recipients were subjected to high protozoan predation pressure and limited nutrient levels, leading to substantial mortality among plasmid-hosting and recipient cells and subsequent decline in the absolute abundance of RP4 plasmids. There was no significant difference between trophozoites and cysts, possibly due to adequate food supply, which facilitate the rapid excystation of protozoa. In terms of absolute abundance, protozoan predation may mitigate the spread of antibiotic plasmids by regulating bacterial biomass and influencing cell growth rates.

Though the copies of RP4 decreased under predation pressure, both *C. steinii* and *A. castellanii* trophozoites facilitated the conjugation frequency of plasmid-mediated ARGs and the expression of conjugation genes in soil bacterial communities. In the presence of protozoa, conjugation frequency reached 10^−2^ (flow cytometry) or even 10^−1^ (qPCR), which were ~1.5 ~ 4.5-fold (*C. steinii*) and 2.7 ~ 5-fold (*A. castellanii*) higher than that in the absence of protozoa. At the molecular level, *C. steinii* enhanced the expression of the *trbB, trfA*, and *traJ* genes, which are responsible for activating the transcription of the key regulator tra that initiates bacterial conjugation, developing the Mpf conjugative channel that allows plasmid DNA to transfer across membranes [46] and regulating plasmid replication initiation [47]. A previous study demonstrated a similar increase in the transfer of plasmid pRT733 from *E. coli* SM10λ + to ciprofloxacin-resistant *E. coli* in the presence of ciliates (*Tetrahymena*), but with a 20-fold higher conjugation frequency (2.2 × 10^−6^) than that in the protozoa-free group (10^−7^) [48]. This discrepancy may be attributed to variations in donor strains [49, 50], composition of microbial communities [51], plasmid type, and environmental conditions that can affect conjugation frequency [51–53]. The effect size of protozoa predation on plasmid transfer frequency is comparable to that of other biotic and abiotic factors. For example, the triclosan-mediated and phthalate-mediated conjugative transfer ratio of RP4 showed a 2.5-fold and 3.82-fold increase when compared with the control groups, respectively [54, 55]. Conjugation ratios in the gut of *Caenorhabditis elegans* were at least two orders of magnitude higher than those in the surrounding soil [47]. This highlights the role of protozoa in plasmid transfer and the dissemination of antimicrobial resistance, thus requiring a deeper understanding of the mechanisms underlying this effect. Indeed, the conjugation frequency, as measured here, is not realized in situ in soil systems, as several biotic and abiotic factors have been shown to determine the success of horizontal plasmid transfer, plasmid persistence and protozoa [56, 57]. Therefore, further research that considers multiple factors in the real environment is needed to gain a comprehensive understanding of the role of protozoa in the persistence and spread of antimicrobial resistance plasmids.

**Figure 6:**
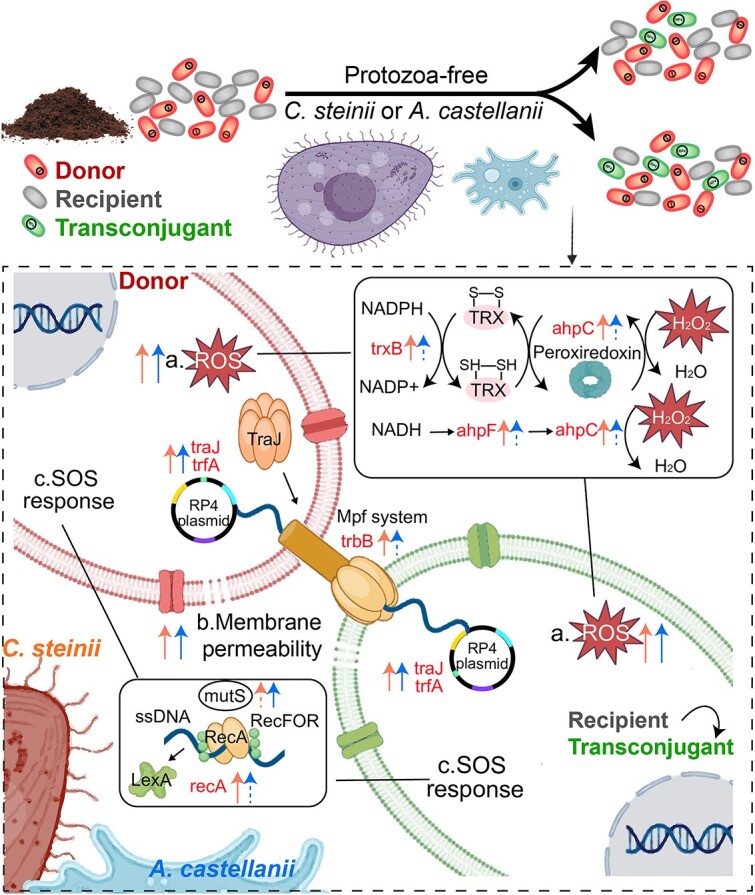
A model depicting the mechanisms underlying the RP4 plasmid-mediated conjugation promoted by *C. steinii* and *A. castellanii,* including (A) increased ROS level, (B) increased membrane permeability, and (C) ROS-, SOS-, and conjugative-related genes expression regulation. Solid and dashed arrows indicate significant and non-significant changes, respectively.

Our study provided evidence that protozoa-facilitated conjugation frequency was associated with ROS levels, membrane permeability, and SOS response. Under predation pressure, both the donor strains and recipients may trigger stress responses that lead to increased ROS production. Increased ROS production is also an effective defensive mechanism used by certain bacteria against protozoan predation, such as *Vibrio vulnificus* RtxA1 [58] and *Vibrio cholerae* biofilms [59]. Elevated ROS and protozoan predation may further disrupt bacterial membranes, promoting the transfer of DNA and potentially enhancing HGT [60, 61]. To protect bacteria from ROS damage and maintain redox balance, the expression of antioxidant defense genes responsible for reducing organic hydroperoxides such as alkyl hydroperoxide reductase genes (*ahpC* and *ahpF*) [62] and thioredoxin reductase (*trxB*) [63] was therefore increased in protozoa group (Fig. 6). Excessive ROS production also triggers SOS response, supported by the increasing expression of gene *mutS, lexA,* and *recA*. SOS response plays a key role not only in DNA repair regulation and gene recombination, [64] but also in conjugative transfer of ARGs and VFs [64, 65]. For instance, the SOS response can induce ARGs encoded by the integrative and conjugative elements SXT of *Vibrio cholerae* [66] and promote the expression of the *qnr* gene*,* a plasmid-borne quinolone resistance determinant in Enterobacteriaceae [67]. Additionally, it regulates class I integron integrases, which are crucial in the HGT of ARGs [68]. Thus, activating the stress response in the soil bacterial community is one of the essential ways for protozoan predation to increase the frequency of plasmid-mediated conjugation. In addition, previous studies reported that predation pressure may also influence plasmid transfer by maintaining bacterial populations in a growth phase [69] or stimulating bacterial adaptive mechanisms, such as gene transfer or biofilm formation [70].

Another important aspect of our study was that different protozoa selected for specific taxa and molecular characteristics of transconjugant communities and altered host range. We found that more than half of the transconjugant ASVs in each treatment were unique and they were subdominant (total abundance less than 1%). Protozoan predation was reported to enhance the growth of subdominant species by increasing complementarity and evenness among bacterial species [16]. The rare biosphere, as an important source of antibiotic resistant plasmids, plays an important part in the persistence and spread of ARGs [71]. Exposure to protozoa reduced the proportion of potentially pathogenic transconjugants, whereas selected for taxa as endosymbionts of protozoa or resistant to grazing, such as *Desulfobacter*, *Reyranella*, *Rhodoplanes*, *Aquabacterium*, *Pantoea*, and *Legionella, d*etected exclusively in protozoan transconjugants [72–77]. Another example is that *Patescibacteria* and *Aquabacterium* were selectively enriched as biomarker transconjugants in the presence of *C. steinii* and *A. castellanii*, respectively. *Patescibacteria*, with their ultra-small cells, limited genome size, and restricted metabolic capacities, are likely dependent on other organisms, such as protists, for survival [78, 79]. *Aquabacterium* can resist phagocytosis through form morphotypes [72]. Moreover, some unique VFs carried by transconjugants in protozoa groups, such as *TlyC* and *Cya*, may also aid bacterial defense against predation*.* The *TlyC* gene has been reported to mediate phagosomal escape, allowing *Salmonella enterica* escaping from endosomal vacuoles into the host cell cytosol [80]. This mechanism may also enhance bacterial defense against amoebic predation, as both macrophages and amoebae share similar intracellular environments and bacterial killing strategies. *Cya*, the main pXO1-encoded toxin gene, is dispensable for the growth of *Bacillus anthracis* within amoebas [81]. Under predation pressure from two protozoa, the host range and molecular characteristics of transconjugants differed. This difference may be due to different feeding rates and selective predation between *C. steinii* and *A. castellanii*, which might affect quorum sensing in microbial communities and enable bacteria to produce compounds, such as VFs to defend against predation [82]. These implied that protozoa may shape the molecular characterization of soil resistomes by enriching VFs capable of resisting protozoan predation. This may be associated with endosymbiosis and predation-resistance.

Taken together, this study demonstrates that protozoa predation reduces the absolute abundance of plasmids whereas enhancing the frequency of plasmid-mediated ARG transfer and conjugation-related gene expression by triggering stress responses of soil microbial communities. Protozoa predation also influences the host range and molecular characteristics of transconjugant communities by selecting taxa potentially resistant to predation or endosymbionts. These findings underscore the crucial role of soil protozoa in driving the dissemination of antibiotic resistance within bacterial communities and further highlight the potential of microbial ecological strategies, such as employing protozoa as microbial agents, to mitigate the rising threat of ARG proliferation from environmental sources.

## Supplementary Material

SI_1_11_wraf009

SI_wraf009

## Data Availability

All raw sequencing data were deposited to the Sequence Read Archive (SRA) under the number PRJNA1108910.

## References

[1] Collaborators AR . Global burden of bacterial antimicrobial resistance in 2019: a systematic analysis. *Lancet* 2022;399:629–55. 10.1016/S0140-6736(21)02724-0 35065702 PMC8841637

[2] Xie S-T, Ding L-J, Huang F-Y et al. VFG-Chip: a high-throughput qPCR microarray for profiling virulence factor genes from the environment. *Environ Int* 2023;172:107761. 10.1016/j.envint.2023.107761 36682204

[3] Liang J, Mao G, Yin X et al. Identification and quantification of bacterial genomes carrying antibiotic resistance genes and virulence factor genes for aquatic microbiological risk assessment. *Water Res* 2020;168:115160. 10.1016/j.watres.2019.115160 31614233

[4] von Wintersdorff CJ, Penders J, van Niekerk JM et al. Dissemination of antimicrobial resistance in microbial ecosystems through horizontal gene transfer. *Front Microbiol* 2016;7:173. 10.3389/fmicb.2016.00173 26925045 PMC4759269

[5] Lerner A, Matthias T, Aminov R. Potential effects of horizontal gene exchange in the human gut. *Front Immunol* 2017;8:1–14. 10.3389/fimmu.2017.01630 29230215 PMC5711824

[6] Castaneda-Barba S, Top EM, Stalder T. Plasmids, a molecular cornerstone of antimicrobial resistance in the one health era. *Nat Rev Microbiol.* 2024;22:18–32. 10.1038/s41579-023-00926-x 37430173 PMC12440250

[7] D'Costa VM, King CE, Kalan L et al. Antibiotic resistance is ancient. *Nature* 2011;477:457–61. 10.1038/nature10388 21881561

[8] Yang K, Xu F, Zhu L et al. An isotope-labeled single-cell raman spectroscopy approach for tracking the physiological evolution trajectory of bacteria toward antibiotic resistance. *Angew Chem Int Ed Engl* 2023;62:e202217412. 10.1002/anie.202217412 36732297

[9] Pepper IL, Brooks JP. Chapter 25- soil microbial influences on “one health”. In: Gentry TJ, Fuhrmann JJ, Zuberer DA (eds), Principles and Applications of Soil Microbiology. Amsterdam, Elsevier, 2021, 681–700. 10.1016/B978-0-12-820202-9.00025-3

[10] Zhu Y-G, Zhao Y, Zhu D et al. Soil biota, antimicrobial resistance and planetary health. *Environ Int* 2019;131:105059. 10.1016/j.envint.2019.105059 31374443

[11] Bahram M, Hildebrand F, Forslund SK et al. Structure and function of the global topsoil microbiome. *Nature* 2018;560:233–7. 10.1038/s41586-018-0386-6 30069051

[12] Nguyen BT, Chen QL, He JZ et al. Microbial regulation of natural antibiotic resistance: understanding the protist-bacteria interactions for evolution of soil resistome. *Sci Total Environ* 2020;705:135882. 10.1016/j.scitotenv.2019.135882 31818598

[13] Rahman MH, Mahbub KR, Espinoza-Vergara G et al. Protozoal food vacuoles enhance transformation in *Vibrio cholerae* through SOS-regulated DNA integration. *ISME J.* 2022;16:1993–2001. 10.1038/s41396-022-01249-0 35577916 PMC9296650

[14] Cairns J, Jalasvuori M, Ojala V et al. Conjugation is necessary for a bacterial plasmid to survive under protozoan predation. *Biol Lett* 2016;12:20150953. 10.1098/rsbl.2015.0953 26843557 PMC4780553

[15] Matsushita M, Okubo T, Hasegawa T et al. *Tetrahymena* promotes interactive transfer of carbapenemase gene encoded in plasmid between fecal *Escherichia coli* and environmental *Aeromonas caviae*. *Microbiol Immunol* 2018;62:720–8. 10.1111/1348-0421.12656 30357893

[16] Saleem M, Fetzer I, Dormann CF et al. Predator richness increases the effect of prey diversity on prey yield. *Nat Commun* 2012;3:1305. 10.1038/ncomms2287 23250435

[17] Shu L, He Z, Guan X et al. A dormant amoeba species can selectively sense and predate on different soil bacteria. *Funct Ecol* 2021;35:1708–21. 10.1111/1365-2435.13824

[18] Musovic S, Dechesne A, Sorensen J et al. Novel assay to assess permissiveness of a soil microbial community toward receipt of mobile genetic elements. *Appl Environ Microbiol* 2010;76:4813–8. 10.1128/AEM.02713-09 20511430 PMC2901734

[19] Klümper U, Riber L, Dechesne A et al. Broad host range plasmids can invade an unexpectedly diverse fraction of a soil bacterial community. *ISME J.* 2015;9:934–45. 10.1038/ismej.2014.191 25333461 PMC4817699

[20] Espinoza-Vergara G, Noorian P, Silva-Valenzuela CA et al. *Vibrio cholerae* residing in food vacuoles expelled by protozoa are more infectious in vivo. *Nat Microbiol* 2019;4:2466–74. 10.1038/s41564-019-0563-x 31570868 PMC7071789

[21] Lin C, Li W-J, Li L-J et al. Movement of protistan trophic groups in soil-plant continuums. *Environ Microbiol* 2023;25:2641–52. 10.1111/1462-2920.16477 37547979

[22] Li L-J, Lin C, Huang X-R et al. Characterizing potential pathogens from intracellular bacterial community of protists in wastewater treatment plants. *Environ Int* 2023;171:107723. 10.1016/j.envint.2022.107723 36584423

[23] Bolyen E, Rideout JR, Dillon MR et al. Reproducible, interactive, scalable and extensible microbiome data science using QIIME 2. *Nat Biotechnol* 2019;37:852–7. 10.1038/s41587-019-0209-9 31341288 PMC7015180

[24] Quast C, Pruesse E, Yilmaz P et al. The SILVA ribosomal RNA gene database project: improved data processing and web-based tools. *Nucleic Acids Res* 2012;41:D590–6. 10.1093/nar/gks1219 23193283 PMC3531112

[25] Chen QL, Li H, Zhou XY et al. An underappreciated hotspot of antibiotic resistance: the groundwater near the municipal solid waste landfill. *Sci Total Environ* 2017;609:966–73. 10.1016/j.scitotenv.2017.07.164 28783909

[26] Andrews S . FastQC: A Quality Control Tool for High Throughput Sequence Data, Cambridge: Babraham Bioinformatics, 2010. http://www.bioinformatics.babraham.ac.uk/projects/fastqc/.

[27] Kanehisa M, Furumichi M, Tanabe M et al. KEGG: new perspectives on genomes, pathways, diseases and drugs. *Nucleic Acids Res* 2016;45:D353–61. 10.1093/nar/gkw1092 27899662 PMC5210567

[28] Yin X, Jiang X, Chai B et al. ARGs-OAP v2.0 with an expanded SARG database and hidden Markov models for enhancement characterization and quantification of antibiotic resistance genes in environmental metagenomes. *Bioinformatics* 2018;34:2263–70. 10.1093/bioinformatics/bty053 29408954

[29] Li D, Luo R, Liu C-M et al. MEGAHIT v1.0: a fast and scalable metagenome assembler driven by advanced methodologies and community practices. *Methods* 2016;102:3–11. 10.1016/j.ymeth.2016.02.020 27012178

[30] Gurevich A, Saveliev V, Vyahhi N et al. QUAST: quality assessment tool for genome assemblies. *Bioinformatics* 2013;29:1072–5. 10.1093/bioinformatics/btt086 23422339 PMC3624806

[31] Hyatt D, Chen GL, Locascio PF et al. Prodigal: prokaryotic gene recognition and translation initiation site identification. *BMC bioinformatics* 2010;11:119. 10.1186/1471-2105-11-119 20211023 PMC2848648

[32] Steinegger M, Söding J. MMseqs2 enables sensitive protein sequence searching for the analysis of massive data sets. *Nat Biotechnol* 2017;35:1026–8. 10.1038/nbt.3988 29035372

[33] Liu B, Zheng D, Jin Q et al. VFDB 2019: a comparative pathogenomic platform with an interactive web interface. *Nucleic Acids Res* 2019;47:D687–92. 10.1093/nar/gky1080 30395255 PMC6324032

[34] Liu M, Xb L, Xie YZ et al. ICEberg 2.0: an updated database of bacterial integrative and conjugative elements. *Nucleic Acids Res* 2018;47:D660–5. 10.1093/nar/gky1123 PMC6323972 30407568

[35] Wang Y, Lu J, Mao L et al. Antiepileptic drug carbamazepine promotes horizontal transfer of plasmid-borne multi-antibiotic resistance genes within and across bacterial genera. *ISME J.* 2019;13:509–22. 10.1038/s41396-018-0275-x 30291330 PMC6331567

[36] Pu Q, Fan XT, Li H et al. Cadmium enhances conjugative plasmid transfer to a fresh water microbial community. *Environ Pollut* 2021;268:115903. 10.1016/j.envpol.2020.115903 33120155

[37] Pu Q, Fan XT, Sun AQ et al. Co-effect of cadmium and iron oxide nanoparticles on plasmid-mediated conjugative transfer of antibiotic resistance genes. *Environ Int* 2021;152:106453. 10.1016/j.envint.2021.106453 33798824

[38] Oksanen J, Blanchet FG, Friendly M et al. R Package ‘Vegan’ Version 2.5–1, CRAN, 2018. https://CRAN.R-project.org/package=vegan.

[39] Zhou S-Y-D, Zhang Q, Neilson R et al. Vertical distribution of antibiotic resistance genes in an urban green facade. *Environ Int* 2021;152:106502. 10.1016/j.envint.2021.106502 33721724

[40] Yang X, Jiang G, Zhang Y et al. MBPD: a multiple bacterial pathogen detection pipeline for one health practices. *iMeta* 2023;2:e82. 10.1002/imt2.82 38868336 PMC10989770

[41] Yang K, Chen Q-L, Chen M-L et al. Temporal dynamics of antibiotic resistome in the plastisphere during microbial colonization. *Environ Sci Technol* 2020;54:11322–32. 10.1021/acs.est.0c04292 32812755

[42] Segata N, Izard J, Waldron L et al. Metagenomic biomarker discovery and explanation. *Genome Biol* 2011;12:R60. 10.1186/gb-2011-12-6-r60 21702898 PMC3218848

[43] Louca S, Parfrey LW, Doebeli M. Decoupling function and taxonomy in the global ocean microbiome. *Science* 2016;353:1272–7. 10.1126/science.aaf4507 27634532

[44] Douglas GM, Maffei VJ, Zaneveld JR et al. PICRUSt2 for prediction of metagenome functions. *Nat Biotechnol* 2020;38:685–8. 10.1038/s41587-020-0548-6 32483366 PMC7365738

[45] Nguyen BT, Bonkowski M, Dumack K et al. Protistan predation selects for antibiotic resistance in soil bacterial communities. *ISME J* 2023;17:2182–9. 10.1038/s41396-023-01524-8 37794244 PMC10689782

[46] Wang H, Qi H, Zhu M et al. MoS_2_ decorated nanocomposite: Fe_2_O_3_@MoS_2_ inhibits the conjugative transfer of antibiotic resistance genes. *Ecotoxicology* 2019;186:109781. 10.1016/j.ecoenv.2019.109781 31622879

[47] Zhou GW, Zheng F, Fan XT et al. Host age increased conjugal plasmid transfer in gut microbiota of the soil invertebrate *Caenorhabditis elegans*. *J Hazard Mater* 2022;424:127525. 10.1016/j.jhazmat.2021.127525 34879519

[48] Matsuo J, Oguri S, Nakamura S et al. Ciliates rapidly enhance the frequency of conjugation between *Escherichia coli* strains through bacterial accumulation in vesicles. *Res Microbiol* 2010;161:711–9. 10.1016/j.resmic.2010.07.004 20691258

[49] Heß S, Kneis D, Virta M et al. The spread of the plasmid RP4 in a synthetic bacterial community is dependent on the particular donor strain. *FEMS Microbiol Ecol* 2021;97:fiab147. 10.1093/femsec/fiab147 34788805

[50] Heß S, Hiltunen T, Berendonk TU et al. High variability of plasmid uptake rates in *Escherichia coli* isolated from sewage and river sediments. *PLoS One* 2020;15:e0232130. 10.1371/journal.pone.0232130 32353032 PMC7192377

[51] Seoane J, Yankelevich T, Dechesne A et al. An individual-based approach to explain plasmid invasion in bacterial populations. *FEMS Microbiol Ecol* 2011;75:17–27. 10.1111/j.1574-6941.2010.00994.x 21091520

[52] Benz F, Huisman JS, Bakkeren E et al. Plasmid- and strain-specific factors drive variation in ESBL-plasmid spread in vitro and in vivo. *ISME J* 2021;15:862–78. 10.1038/s41396-020-00819-4 33149210 PMC8026971

[53] Jong MC, Harwood CR, Blackburn A et al. Impact of redox conditions on antibiotic resistance conjugative gene transfer frequency and plasmid fate in wastewater ecosystems. *Environ Sci Technol* 2020;54:14984–93. 10.1021/acs.est.0c03714 33191749

[54] Lu J, Yu Z, Ding P et al. Triclosan promotes conjugative transfer of antibiotic resistance genes to opportunistic pathogens in environmental microbiome. *Environ Sci Technol* 2022;56:15108–19. 10.1021/acs.est.2c05537 36251935

[55] Wu J, Zhou JH, Liu DF et al. Phthalates promote dissemination of antibiotic resistance genes: an overlooked environmental risk. *Environ Sci Technol* 2023;57:6876–87. 10.1021/acs.est.2c09491 37083356

[56] Yang QE, Ma X, Li M et al. Evolution of triclosan resistance modulates bacterial permissiveness to multidrug resistance plasmids and phages. *Nat Commun* 2024;15:3654. 10.1038/s41467-024-48006-9 38688912 PMC11061290

[57] San Millan A, Peña-Miller R, Toll-Riera M et al. Positive selection and compensatory adaptation interact to stabilize non-transmissible plasmids. *Nat Commun* 2014;5:5208. 10.1038/ncomms6208 25302567 PMC4208098

[58] Chung K-J, Cho E-J, Kim MK et al. RtxA1-induced expression of the small GTPase Rac2 plays a key role in the pathogenicity of *Vibrio vulnificus*. *J Inf Secur* 2010;201:97–105. 10.1086/648612 19919301

[59] Noorian P, Hu J, Chen Z et al. Pyomelanin produced by *Vibrio cholerae* confers resistance to predation by *Acanthamoeba castellanii*. *FEMS Microbiol Ecol* 2017;93:fix147. 10.1093/femsec/fix147 29095994 PMC5812506

[60] Qiu Z, Yu Y, Chen Z et al. Nanoalumina promotes the horizontal transfer of multiresistance genes mediated by plasmids across genera. *Proc Natl Acad Sci USA* 2012;109:4944–9. 10.1073/pnas.1107254109 22411796 PMC3323979

[61] Suzuki S, Sano D. Effect of protists on horizontal transfer of antimicrobial resistance genes in water environment. *J Water Environ* 2023;21:97–107. 10.2965/jwet.22-095

[62] Imlay JA . The molecular mechanisms and physiological consequences of oxidative stress: lessons from a model bacterium. *Nat Rev Microbiol* 2013;11:443–54. 10.1038/nrmicro3032 23712352 PMC4018742

[63] Lu J, Holmgren A. The thioredoxin antioxidant system. *Free Radic Biol Med* 2014;66:75–87. 10.1016/j.freeradbiomed.2013.07.036 23899494

[64] Baharoglu Z, Mazel D. SOS, the formidable strategy of bacteria against aggressions. *FEMS Microbiol Rev* 2014;38:1126–45. 10.1111/1574-6976.12077 24923554

[65] Úbeda C, Maiques E, Knecht E et al. Antibiotic-induced SOS response promotes horizontal dissemination of pathogenicity island-encoded virulence factors in *Staphylococci*. *Mol Microbiol* 2005;56:836–44. 10.1111/j.1365-2958.2005.04584.x 15819636

[66] Beaber JW, Hochhut B, Waldor MK. SOS response promotes horizontal dissemination of antibiotic resistance genes. *Nature* 2004;427:72–4. 10.1038/nature02241 14688795

[67] Da Re S, Garnier F, Guérin E et al. The SOS response promotes *qnrB* quinolone-resistance determinant expression. *EMBO Rep* 2009;10:929–33. 10.1038/embor.2009.99 19556999 PMC2726673

[68] Guerin É, Cambray G, Sanchez-Alberola N et al. The SOS response controls integron recombination. *Science* 2009;324:1034–4. 10.1126/science.1172914. 19460999

[69] Kotilainen MM, Grahn AM, Bamford JK et al. Binding of an *Escherichia coli* double-stranded DNA virus PRD1 to a receptor coded by an IncP-type plasmid. *J Bacteriol* 1993;175:3089–95. 10.1128/jb.175.10.3089-3095.1993 8387995 PMC204630

[70] Blom JF, Horňák K, Simek K et al. Aggregate formation in a freshwater bacterial strain induced by growth state and conspecific chemical cues. *Environ Microbiol* 2010;12:2486–95. 10.1111/j.1462-2920.2010.02222.x 20406293

[71] Loftie-Eaton WCA, Perry D. Contagious antibiotic resistance: plasmid transfer among bacterial residents of the zebrafish gut. *Appl Environ Microbiol* 2021;87:e02735–20. 10.1128/AEM.02735-20 33637574 PMC8091013

[72] Åberg OZ . Exploring the Anaerobic Protist Anaeramoeba Flamelloides: Culturing Methodology, Cell Structure Imaging, Antibiotic Assay and Symbiont Genomics. Sweden: Uppsala Universitet, 2024, Masters thesis,.

[73] Pagnier I, Croce O, Robert C et al. Genome sequence of *Reyranella massiliensis*, a bacterium associated with amoebae. *J Bacteriol* 2012;194:5698. 10.1128/JB.01260-12 23012280 PMC3458673

[74] Amaro F, Wang W, Gilbert JA et al. Diverse protist grazers select for virulence-related traits in *Legionella*. *ISME J.* 2015;9:1607–18. 10.1038/ismej.2014.248 25575308 PMC4478701

[75] Thomas V, Herrera-Rimann K, Blanc DS et al. Biodiversity of amoebae and amoeba-resisting bacteria in a hospital water network. *Appl Environ Microbiol* 2006;72:2428–38. 10.1128/AEM.72.4.2428-2438.2006 16597941 PMC1449017

[76] Michaela MS, Jakob P, Roland P et al. Succession of bacterial grazing defense mechanisms against protistan predators in an experimental microbial community. *Aquat Microb Ecol* 2005;38:215–29. 10.3354/ame038215

[77] Smith Derek DN, Nickzad A, Déziel E et al. A novel glycolipid biosurfactant confers grazing resistance upon *Pantoea ananatis* BRT175 against the social amoeba *Dictyostelium discoideum*. *mSphere* 2016;1:00075–15. 10.1128/mSphere.00075-15 PMC4863597 27303689

[78] Gios E, Mosley OE, Weaver L et al. Ultra-small bacteria and archaea exhibit genetic flexibility towards groundwater oxygen content, and adaptations for attached or planktonic lifestyles. *ISME Commun* 2023;3:13. 10.1038/s43705-023-00223-x 36808147 PMC9938205

[79] Tian R, Ning D, He Z et al. Small and mighty: adaptation of superphylum Patescibacteria to groundwater environment drives their genome simplicity. *Microbiome* 2020;8:51. 10.1186/s40168-020-00825-w 32252814 PMC7137472

[80] Whitworth T, Popov VL, Yu XJ et al. Expression of the *Rickettsia prowazekii pld* or *tlyC* gene in *Salmonella enterica* serovar Typhimurium mediates phagosomal escape. *Infect Immun* 2005;73:6668–73. 10.1128/IAI.73.10.6668-6673.2005 16177343 PMC1230948

[81] Chen H, Verplaetse E, Jauslin T et al. The fate of bacteria of the *Bacillus cereus* group in the amoeba environment. *Microb Ecol* 2022;83:1088–104. 10.1007/s00248-021-01828-2 34342700

[82] Mukherjee S, Bassler BL. Bacterial quorum sensing in complex and dynamically changing environments. *Nat Rev Microbiol* 2019;17:371–82. 10.1038/s41579-019-0186-5 30944413 PMC6615036

