# Supplementary material for: Protozoa-enhanced conjugation frequency alters the dissemination of soil antibiotic resistance
Source: ISME J. 2025 Jan 27;19(1):wraf009. doi: 10.1093/ismejo/wraf009 (PMC11845867; doi:10.1093/ismejo/wraf009)
Supplement: SI_1_11_wraf009 [file si_1_11_wraf009.docx]

## Supplemental material

**Protozoa-enhanced conjugation frequency alters the dissemination of soil antibiotic resistance**

Chenshuo Lin^1, 2^, Li-Juan Li^1, 2^ Kai Yang^1^, Jia-Yang Xu^1, 3^, Xiao-Ting Fan^1^, Qing-Lin Chen^1, 2^, Yong-Guan Zhu^1, 2, 4^

^1^ State Key Laboratory for Ecological Security of Regions and Cities, Ningbo Urban Environment Observation and Research Station, Institute of Urban Environment, Chinese Academy of Sciences, 1799 Jimei Road, Xiamen 361021, China

^2^ Zhejiang Key Laboratory of Urban Environmental Processes and Pollution Control, CAS Haixi Industrial Technology Innovation Center in Beilun, Ningbo 315830, China.

^3^ University of Chinese Academy of Sciences, 19A Yuquan Road, Beijing 100049, China

^4^ State Key Laboratory of Urban and Regional Ecology, Research Center for Eco-Environmental Sciences, Chinese Academy of Sciences, Beijing 100085, China

### Identification and growth of protozoa

Protozoa *C. steinii* and *A. castellanii* were isolated from the agriculture field in Xiamen, China (24°64′N, 118°05′E). Five grams of soil were shaken with 20 mL sterile Page's amoeba saline (PAS) (120 mg NaCl, 4 mg MgSO_2_⋅7H_2_O, 4 mg CaCl_2_⋅2H_2_O, 142 mg Na_2_HPO_4_, and 136 mg KH_2_PO_4_ in 1 liter of distilled water), for 30 minutes. Soil suspension was then transferred into 96-well plates, which contained sterile Page's amoeba saline and heat-inactivated *Escherichia coli* DH5α. After 3 days of incubation at 30°C, protozoa strains were diluted several times to obtain the pure protozoa. Protozoa were identified via almost complete 18S rRNA gene sequencing with PSSU (5′-CTTTCGATGGTAGTGTATTGGACTAC-3′) and EukB (5′-TGATCCTTCTGCAGGTTCACTAC-3′) primers[1] (Supplementary Table S1). The taxonomic classification of the protist strain was determined by conducting a BLASTn search against the NCBI GenBank database.

The pure protozoa *Colpoda steinii* and *A. castellanii* were routinely cultured using sterile Page's amoeba saline and heat-inactivated *E. coli* DH5α in tissue culture flasks in the dark at 30°C. Prior to experiments, protozoa were separated from *Escherichia coli* using S3e Cell Sorter with a purity yield mode based on the higher internal complexity (side scatter, SSC) versus bigger cell size (forward scatter, FSC) twice. Subsequently, sorted protozoa were washed three times with PAS and collected by centrifugation at 1000 g for 5 min. Protozoa were enumerated microscopically using a hemocytometer.

### Recipient

After passing through a 2 mm sieve, soil bacteria were extracted using Nycodenz density gradient separation as described previously [2]. Briefly, 15 g soils were homogenized in 10 ml of PBS with 0.5% (v/v) Tween 20 and vortexed vigorously for 30 min. Then, 1 vol of soil suspension was carefully introduced into 1 vol of Nycodenz solution at a density of 1.42 g/ml, followed by centrifugation at 14,000g for 90 min. The upper and middle aqueous layers containing bacteria were collected in PBS. The microbial suspension was filtered sequentially through 5.0 µm and 1 µm membranes to eliminate fauna and protists. An inverted microscope (Nikon Eclipse TiU, Tokyo, Japan) ensured the absence of protozoa. Soil microbial enumeration was conducted using flow cytometry (FlowSight Imaging Flow Cytometer, Amnis Millipore, United States) with SYTO 9 staining.

### qPCR

All qPCR were performed in triplicate using Roche 480 ((Roche Inc., USA). Each qPCR has a 20 μL reaction mixture consisting of 10 μL 2 × LightCycler 480 SYBR Green I Master, 1 μL of each primer, 2 μL of template DNA, 0.5 μL 20 μg/ml of bovine serum albumin and 5.5 μL of nuclease-free water. The thermal cycle for *gfp* consisted of a 5 min of 95°C, followed by 40 cycles of 95°C for 45 s, 58°C for 1 min, 72°C for 1 min, and a final extension at 72°C for 10 min. The thermal cycle for *dsred* consisted of a 10 min of 95°C, followed by 40 cycles of 95°C for 15 s, 60°C for 1 min, 60°C for 2 min, and a final extension at 72°C for 10 min. The thermal cycle for the 16S rRNA gene consisted of 5 min of 95°C, followed by 40 cycles of 95°C for 40 s, 58°C for 30 s, 72°C for 30 s, and a final extension at 72°C for 10 min. The 10-fold serial dilutions of standard plasmids bearing 16S rRNA*, gfp*, and *dsRed* genes were used to generate standard curves.


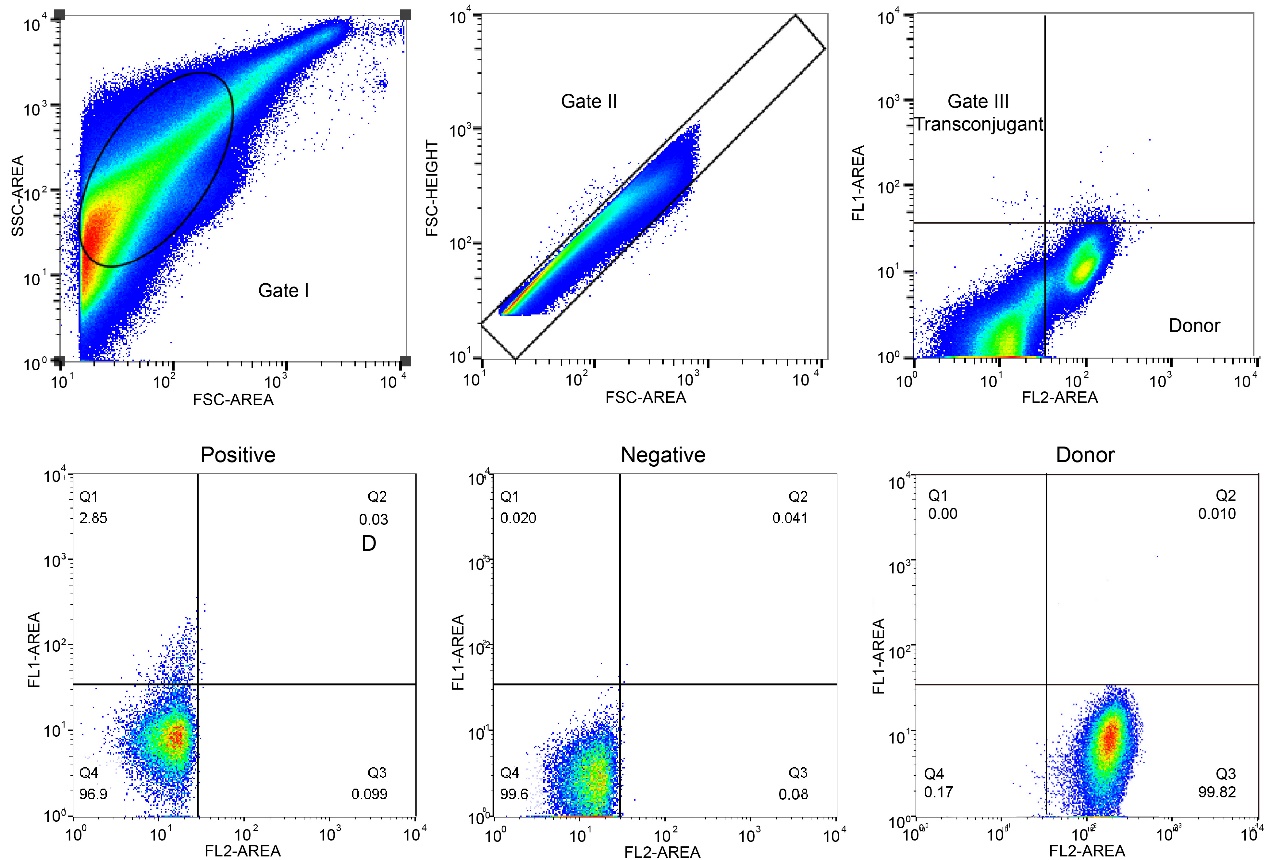


Fig. S1 The gating strategy of FACS sorting for transconjugant cells by flow cytometry. from samples, *P. putida* KT2442 carrying gfp-RP4 plasmid, negative control (*Escherichia coli* K12), and positive control (*E. coli* K12 carrying RP4::*gfp*). Gate I sorts for bacterial size based on forward and side scatter (FSC-Area and SSC-Area); Gate II sorts for single cells according to FSC-Height versus FSC-Area; and Gate III sorts for transconjugant cells with high green fluorescence but no red fluorescence.


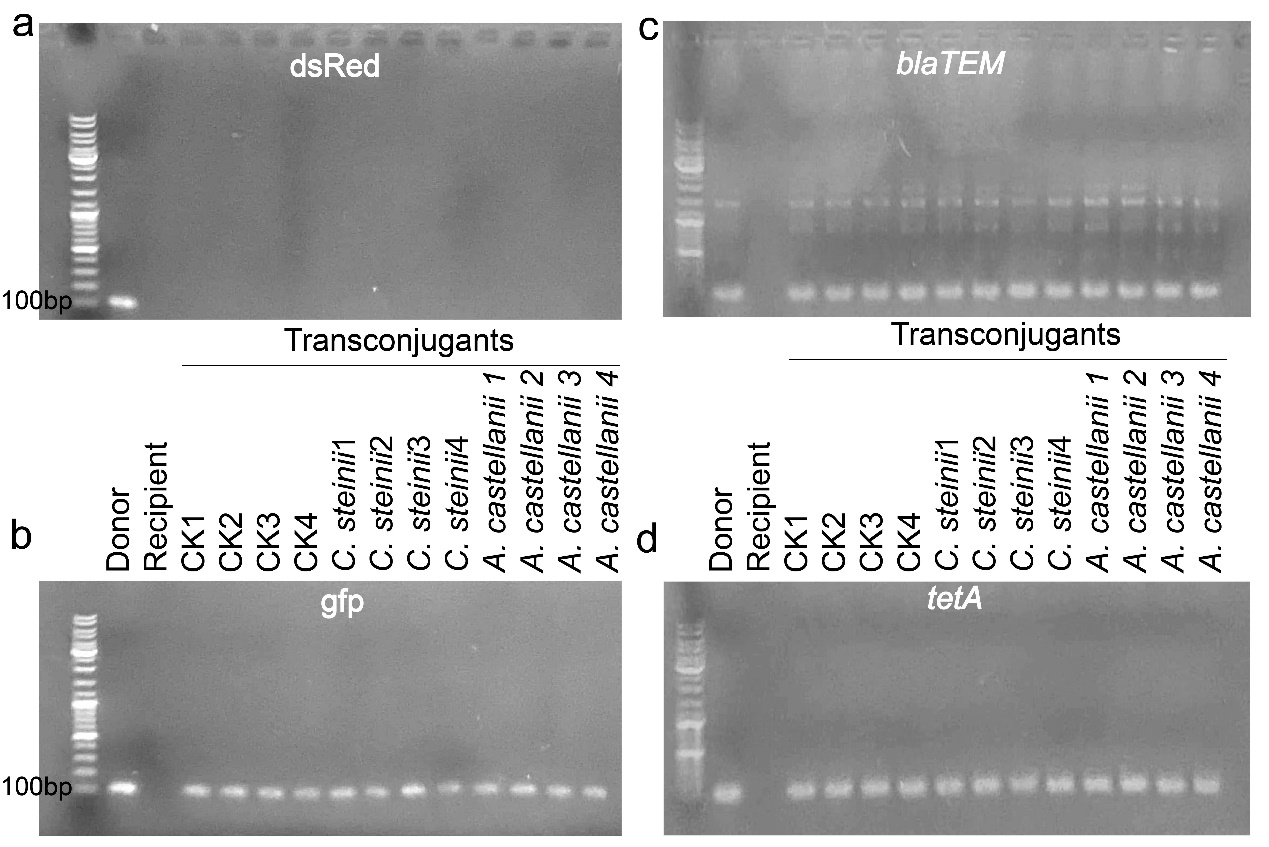


Fig. S2. The gel photos of the gel electrophoresis of PCR on (a) *dsRed*, (b) *gfp*, (c) *blaTEM*, and (d) *tetA* genes on DNA extracted from donor, recipient and transconjugants.


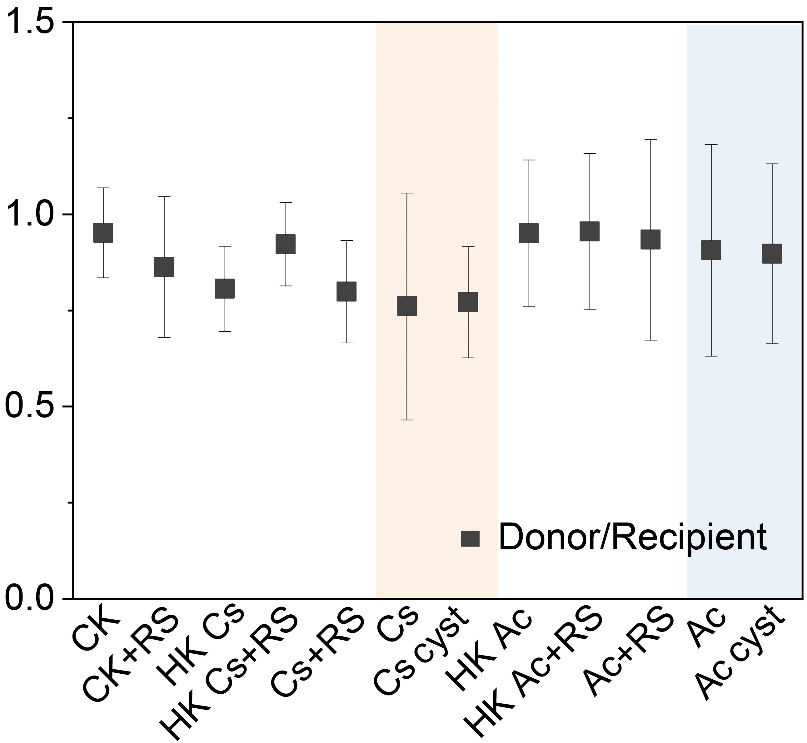


Fig. S3. The ratios of donor to recipient calculated by qPCR results.


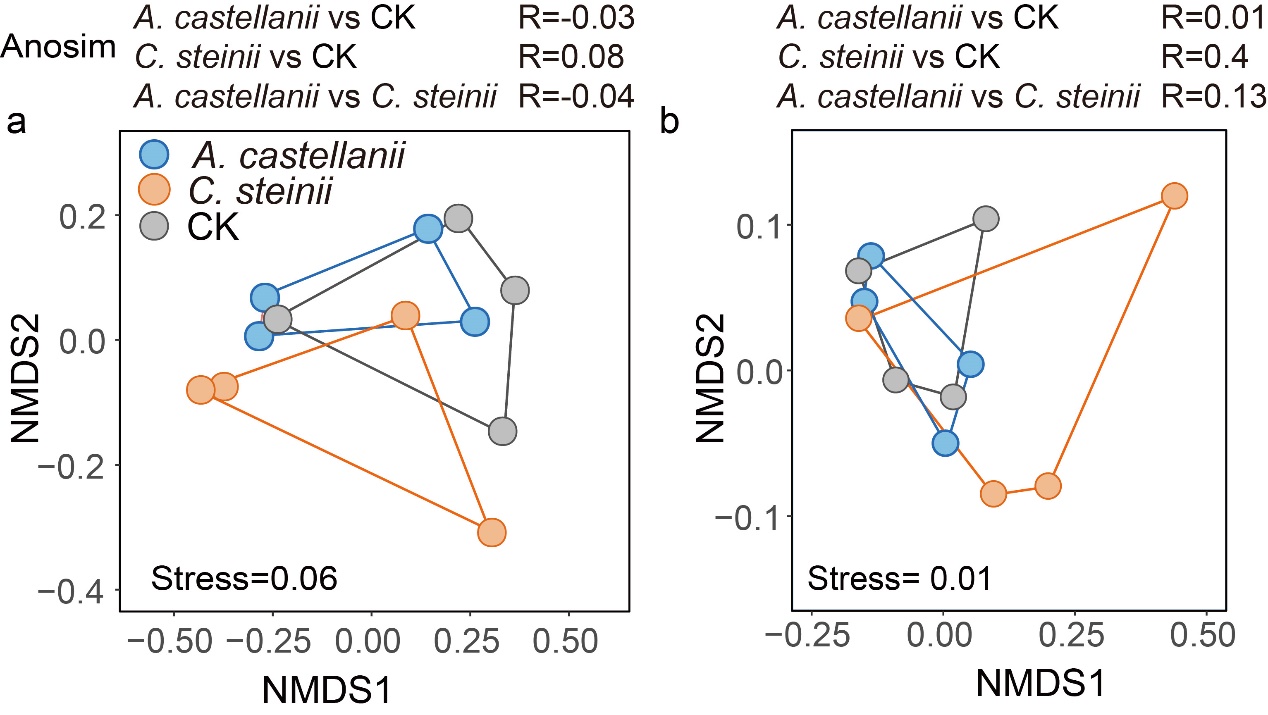


Fig. S4. Nonmetric multidimensional scaling (NMDS) ordinations depicting the distribution patterns of (a) bacterial community structure and (b) Bray–Curtis distance matrices of KEGG Orthology (KO) of different transconjugants. The significant difference were determined by nonparametric Kruskal‑Wallis test.


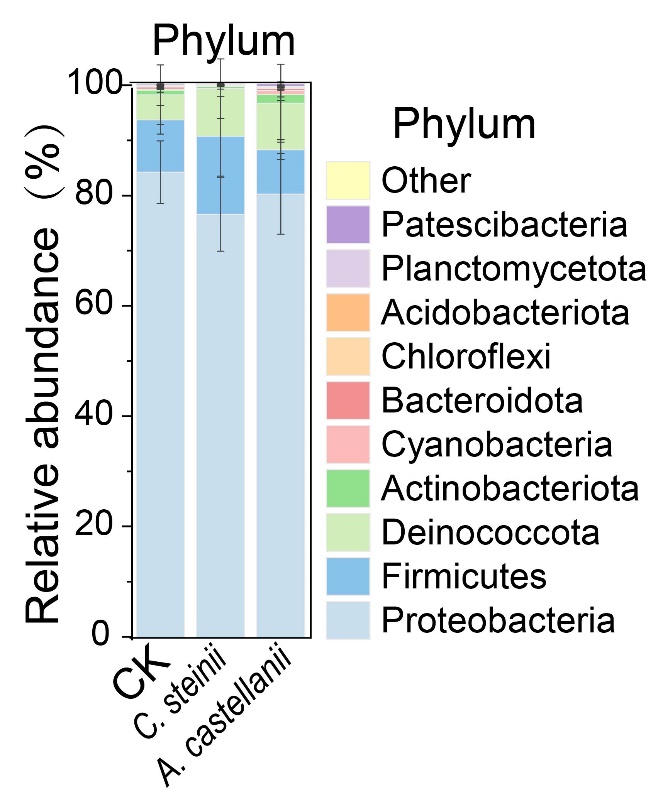


Fig. S5. Microbial community compositions in transconjugant pools at phylum level


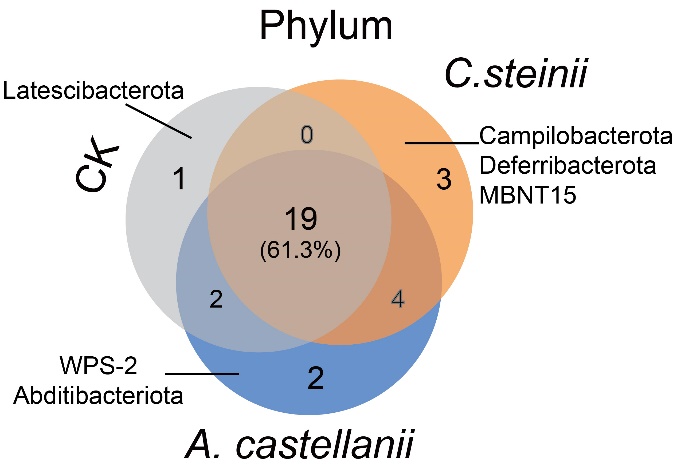


Fig. S6 Venn diagrams showing the number of shared and unique bacterial phylum in different transconjugant pools.


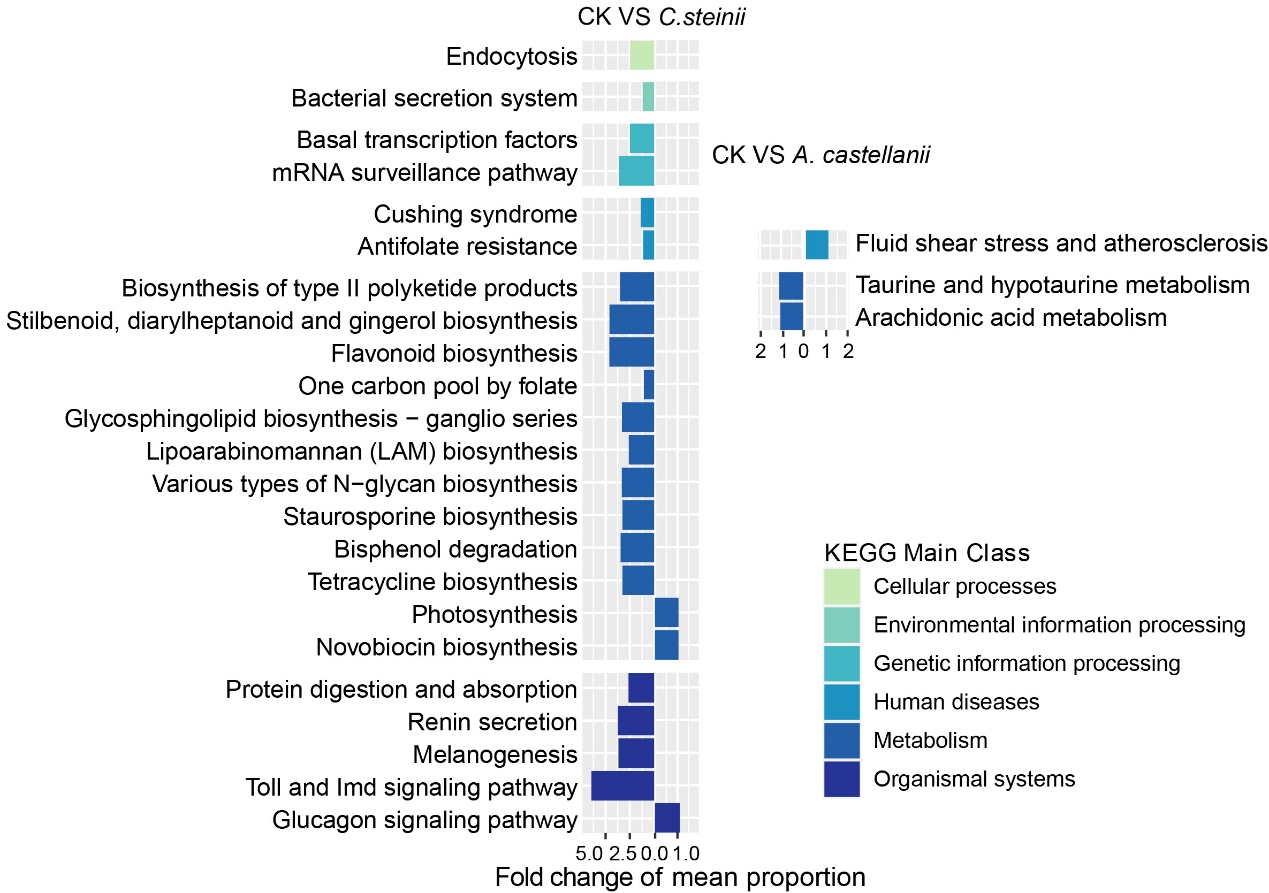


Fig. S7. Potential functional capabilities of transconjugant communities significantly different between protozoa and protozoa-free group by nonparametric Kruskal-Wallis test (level 3 KEGG Orthologies).


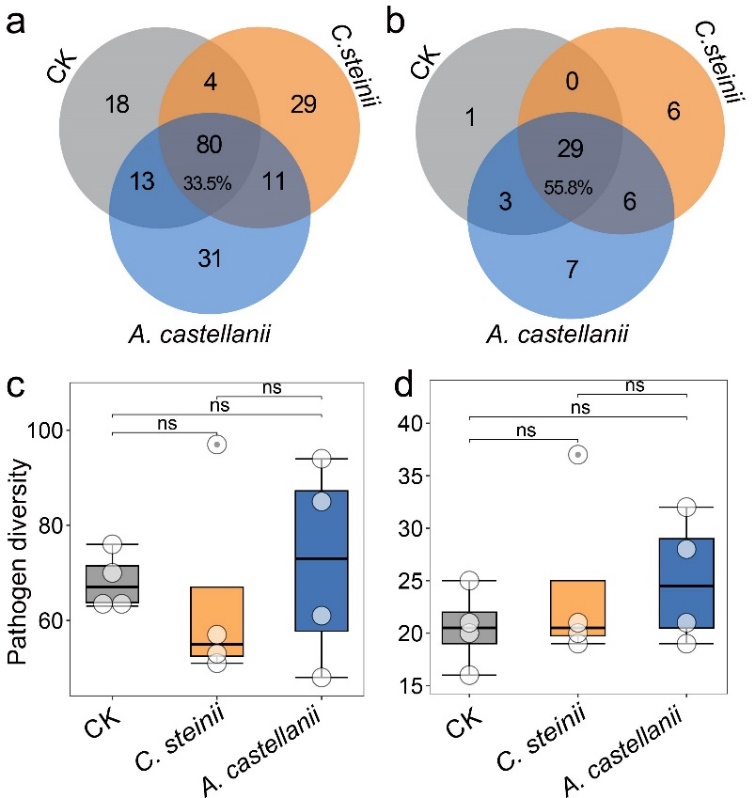


Fig. S8. Venn diagrams showing the number of shared (a) potential pathogens based on MBPD database and (b) self-constructed potential human pathogens, and the diversity of potential pathogenic transconjugant in each group based on (c) MBPD database and (d) self-constructed potential human pathogens.


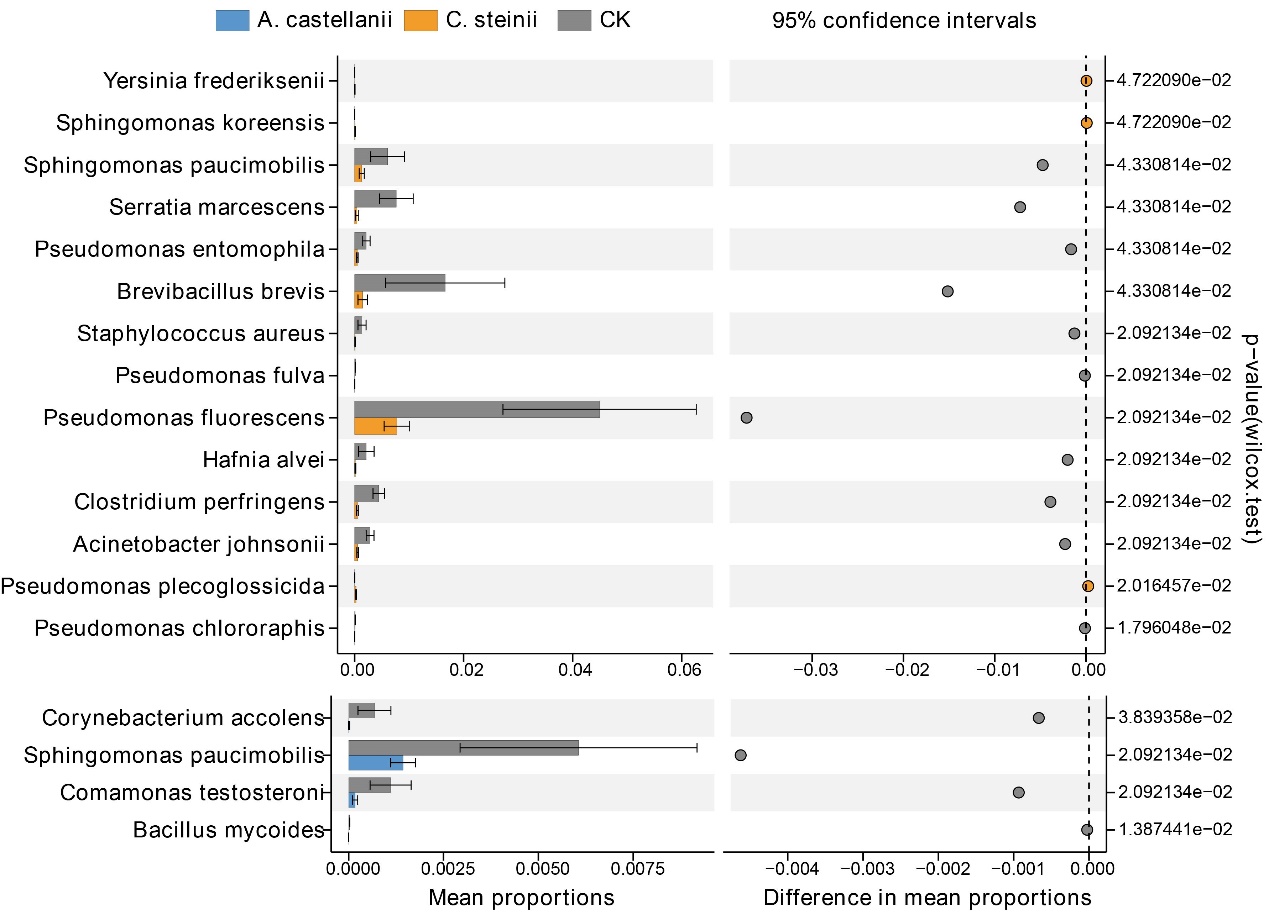


Fig. S9. Potential pathogens statistically different between protozoa-free group (CK) and protozoa (*C. steinii* or *A. castellanii*) groups based on MBPD database.


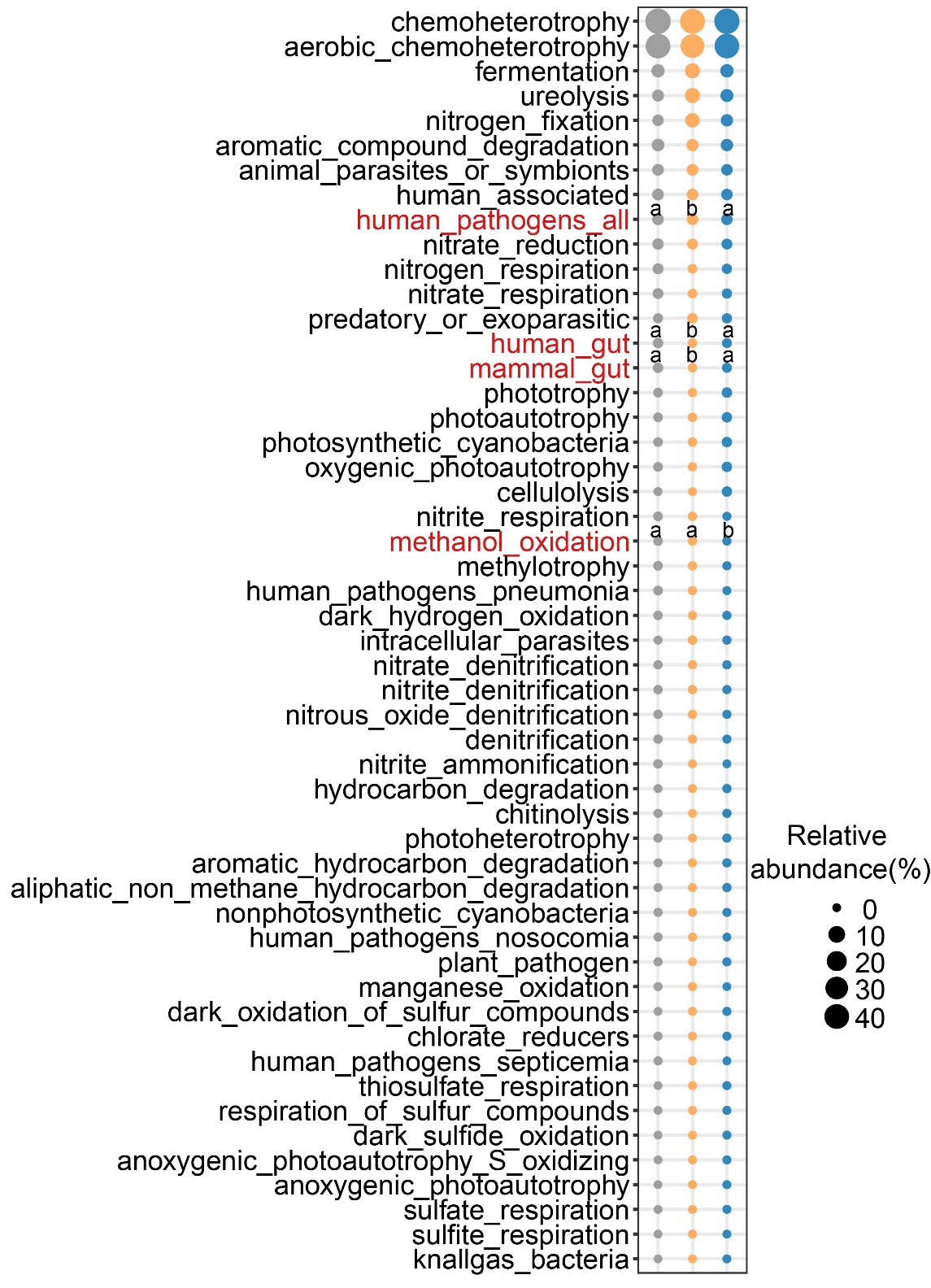


Fig. S10. Functional potentials of bacterial transconjugants. Different letters above bubbles indicate a significant difference determined by nonparametric Kruskal-Wallis test.

Table S1. Primers used in this study for PCR experiment.

| Gene | Primer sequences (5’-3’) | Annealing temperature (°C) |
| --- | --- | --- |
| *gfp* | F：CACTTGTCACTACTTTCGGTTATGG  R：CTTCGGGCATGGCACTCTT | 58 |
| *dsRed* | F：CCTGTTATGCAAAAG AAGACAATG  R：GTAATGACCACCGTCTTTCAG | 58 |
| 16S rRNA | F：GGGTTGCGCTCGTTGC  R：ATGGYTGTCGTCAGCTCGTG | 58 |
| *tetA* | F：CTCACCAGCCTGACCTCGAT  R：CACGTTGTTATAGAAGCCGCATAG | 60 |
| *blaTEM* | F：CGCCGCATACACTATTCTCAG  R：GCTTCATTCAGCTCCGGTTC | 60 |

Table S2 The primers and annealing for RT-PCR

| Gene | Primer sequences (5’-3’) | Annealing temperature (°C) |
| --- | --- | --- |
| *trbB* | F：TAGAAACGGTGGCCGGATTC  R：CGGCGCTTTTAATGACCTCG | 58 |
| *trfA* | F：GAAGCCCATCGCCGTCGCCTGTAG  R：GCCGACGATGACGAACTGGTGTGG | 58 |
| *traJ* | F：CTGCTTCTCTTCGATCTTC  R：CCAGGGCTACAAAATCAC | 56 |
| *ahpC* | F：CTGAAAGGCAAGTGGTCTG  R：GTATTCGATCTTGCCGATG | 56 |
| *ahpF* | F：GGCGTTTTTGTATCCGAGTTT  R：GTCCGCGCTGAAGGTAATG | 58 |
| *trxB* | F：CCTTAATCTTTGCGACCTCGC  R：GACTTCGGTGGTGGTGGTC | 56 |
| *mutS* | F：GCTGTGGGAATTTGAAATCG  R：AGGTACGCTGGGTGTCTTTG | 58 |
| *recA* | F：CCGCAAAACGCGGTATAGTA  R：CCTTCGCCGTACATAATGTC | 58 |
| *lexA* | F：TCAGTGGGATGTCGATGAAA  R：GCCAGACCCTCAATGGTAAA | 58 |

Table S3 Mean relative abundance of potential pathogens in transconjugant communities based on MBPD database and potential human pathogen database

Reference

1. Lin C, Li L-J, Ren K, Zhou S-Y-D, Isabwe A, Yang L-Y et al. Phagotrophic protists preserve antibiotic-resistant opportunistic human pathogens in the vegetable phyllosphere. ISME Commun. 2023;3**:** 94.

2. Cui L, Yang K, Li H-Z, Zhang H, Su J-Q, Paraskevaidi M et al. Functional single-cell approach to probing nitrogen-fixing bacteria in soil communities by resonance Raman spectroscopy with ^15^N_2_ labeling. Anal Chem. 2018;90**:** 5082-5089.
